# FliZ Is a Global Regulatory Protein Affecting the Expression of Flagellar and Virulence Genes in Individual *Xenorhabdus nematophila* Bacterial Cells

**DOI:** 10.1371/journal.pgen.1003915

**Published:** 2013-10-31

**Authors:** Grégory Jubelin, Anne Lanois, Dany Severac, Stéphanie Rialle, Cyrille Longin, Sophie Gaudriault, Alain Givaudan

**Affiliations:** 1 INRA, UMR 1333 Laboratoire DGIMI, Montpellier, France; 2 Université Montpellier 2, UMR 1333 Laboratoire DGIMI, Montpellier, France; 3 MGX-Montpellier GenomiX, c/o IGF-Institut de Génomique Fonctionnelle, Montpellier, France; 4 CEA, Genoscope & CNRS-UMR 8030, Laboratoire d'Analyse Bioinformatique en Génomique et Métabolisme, Evry, France; Genentech, United States of America

## Abstract

Heterogeneity in the expression of various bacterial genes has been shown to result in the presence of individuals with different phenotypes within clonal bacterial populations. The genes specifying motility and flagellar functions are coordinately regulated and form a complex regulon, the flagellar regulon. Complex interplay has recently been demonstrated in the regulation of flagellar and virulence gene expression in many bacterial pathogens. We show here that FliZ, a DNA-binding protein, plays a key role in the insect pathogen, *Xenorhabdus nematophila*, affecting not only hemolysin production and virulence in insects, but efficient swimming motility. RNA-Seq analysis identified FliZ as a global regulatory protein controlling the expression of 278 *Xenorhabdus* genes either directly or indirectly. FliZ is required for the efficient expression of all flagellar genes, probably through its positive feedback loop, which controls expression of the *flhDC* operon, the master regulator of the flagellar circuit. FliZ also up- or downregulates the expression of numerous genes encoding non-flagellar proteins potentially involved in key steps of the *Xenorhabdus* lifecycle. Single-cell analysis revealed the bimodal expression of six identified markers of the FliZ regulon during exponential growth of the bacterial population. In addition, a combination of fluorescence-activated cell sorting and RT-qPCR quantification showed that this bimodality generated a mixed population of cells either expressing (“ON state”) or not expressing (“OFF state”) FliZ-dependent genes. Moreover, studies of a bacterial population exposed to a graded series of FliZ concentrations showed that FliZ functioned as a rheostat, controlling the rate of transition between the “OFF” and “ON” states in individuals. FliZ thus plays a key role in cell fate decisions, by transiently creating individuals with different potentials for motility and host interactions.

## Author Summary

Heterogeneity in the expression of bacterial genes may result in the presence of cells with different phenotypes in an isogenic population. The existence of such “non-genetic individuality” was the first described many years ago for the flagellum-driven swimming behavior of bacteria. In this study, we identified a new bimodal switch controlling the expression of genes involved in flagellum biosynthesis and host interactions in the insect pathogen *Xenorhabdus nematophila*. This switch is modulated by a transcriptional regulator called FliZ. In addition to identifying all the specific genes up- and downregulated by FliZ, we showed that the concentration of FliZ fine-tuned the expression of FliZ target genes, resulting in individuals with different potentials for bacterial locomotion, host colonization and virulence.

## Introduction

Flagella are complex surface structures that serve as the primary means of locomotion in many bacterial species and allow many bacterial pathogens to adhere to and invade cells and in some cases to secrete virulence factors [1]. More than 50 genes are involved in the biogenesis and function of a flagellum in *Escherichia coli* or *Salmonella enterica* serovar Typhimurium (*S.* Typhimurium) [2]. Flagellar gene expression is sequential mirroring the timing of the assembly process [2], [3]. First expressed is the class I operon, *flhDC*, the products of which, FlhD_4_C_2_ heterohexamers, are required for the expression of all other flagellar genes [4]–[6]. The *E. coli* FlhD_4_C_2_ complex activates class II operons, including the structural genes for flagellar hook-basal body components (a type III secretion system) and the alternative sigma factor FliA [7]. The *fliA* gene is the first gene of the *fliAZY* operon in *E. coli* and *S.* Typhimurium and its product, sigma 28, directs transcription of the class III genes encoding the filament protein called flagellin, hook-associated proteins, motor proteins and various chemotaxis proteins [8]. The central channel of the flagellar apparatus is thought to serve as a passage for both flagellar component proteins and for the flagellar regulatory protein FlgM, an anti-sigma-28 factor [5], [9]. Thus, the accumulation of FlgM in the cell due to the prevention of its export blocks the transcription of class III genes, including that encoding flagellin. Two other genes within the flagellar regulon, *fliT* and *fliZ*, have been shown to regulate class II gene transcription in *S.* Typhimurium [10]. Disruption of the *fliT* gene increases class II gene transcription, whereas disruption of the *fliZ* gene decreases class II gene transcription, with no effect on class I transcription, suggesting that FliT and FliZ are negative and positive regulators, respectively. The type III secretion chaperone FliT has been shown to act as an anti-FlhD_4_C_2_ factor preventing the formation of the FlhD_4_C_2_-DNA complex and inhibiting its binding to class II promoters [11].

FliZ is encoded by a gene in the *fliA* operon, and orthologs are found only in the flagellar regulon of members of the family Enterobacteriaceae. The precise mechanism of action of FliZ remains unclear. It has been reported to activate class II flagellar gene expression and there is some evidence that it is involved in the posttranslational regulation of FlhD_4_C_2_ activity in *S.* Typhimurium [12]. However, the FliZ protein contains a region resembling the core DNA-binding domain of phage integrases [13], [14], suggesting that it may play a direct role in the regulation of transcription. Indeed, a primary study in *Xenorhabdus nematophila* (Enterobacteriaceae) showed that FliZ activated the transcription of class II flagellar genes by direct binding to the *flhDC* promoter [13]. Another study showed that FliZ indirectly activated flagellar gene expression in *S.* Typhimurium by binding directly to *nlpC* promoter and repressing the transcription of the associated *ydiV* gene, which encodes an anti-FlhDC factor [15].

Flagellar regulators, such as FliZ, have been implicated in processes other than flagellum synthesis. FliZ has been shown to be an abundant DNA-binding protein that inhibits gene expression mediated by RpoS in *E. coli* by recognizing operator sequences resembling the −10 region of RpoS-dependent promoters [14]. Previous studies have also shown that *fliZ* mutation significantly reduces *hilA* expression and intestinal *S.* Typhimurium colonization in mice [16]. Indeed, indirect regulation by FliZ has been shown to upregulate the expression of the SPI1 type three secretion system (T3SS) in *S.* Typhimurium, where FliZ controls HilD protein activity upstream from the HilC/RtsA/HilA transcriptional cascade [17].

As in *S.* Typhimurium, FliZ has been shown to mediate the coordinate regulation of flagellum synthesis and virulence in the insect pathogen *X. nematophila* [13]. *Xenorhabdus nematophila* displays complex interactions with invertebrates, has a symbiotic lifestyle with nematodes of the genus *Steinernema* and is pathogenic to insect larvae [18], [19]. The successful colonization of two invertebrate hosts requires *Xenorhabdus* to cope with shifting host environments, by interconnecting the various gene networks [19]. Various regulatory proteins of *Xenorhabdus* are involved in host interactions, but it has been shown that the *fliAZ* operon plays a central role in controlling lipase and hemolysin production and in motility and full virulence in insects [13]. Indeed, FliA coordinates the expression of class III flagellar genes, such as the flagellin-encoding gene *fliC*, and the two non-flagellar genes, *xlpA* and *xrtA*, encoding a lipase and a protease, respectively [20], whereas FliZ binds directly to the promoter regions of two different hemolysin-encoding operons, *xaxAB* [21] and *xhlBA* [22], activating the transcription even in the absence of the FlhD_4_C_2_ complex [13]. A real-time analysis of virulence gene expression during insect infection revealed that the expression of FliZ-dependent hemolysin genes coincided with the increase in iron availability detected at the time of insect death, suggesting that iron availability is a signal governing the adaptation of *X. nematophila* to changes in host environments. Interestingly, this study also revealed that the expression of the *fliC* and *xaxAB* genes in *Xenorhabdus* was heterogeneous at the individual cell level [23].

In 1976, Spudich and Koshland reported the existence of “non-genetic individuality” in monitoring the swimming behavior of *S.* Typhimurium at the level of individual cells [24]. More recently, the molecular origin of the temporal variations of chemotaxis system signaling between individual bacteria was reinvestigated, resulting in the demonstration of a role for the relative concentration of a key chemotaxis network component, CheR [25]. Furthermore, high levels of stochastic phenotypic variation have been reported for flagellar genes in *S.* Typhimurium [26]–[28] and *Bacillus subtilis* [29]. We show here that FliZ plays a key role in *Xenorhabdus*, not only in hemolysin activities and full virulence in insects, but also in efficient swimming motility. We demonstrate that efficient expression of the entire flagellar regulon requires the FliZ-dependent positive feedback loop controlling expression of the master operon *flhDC*. We also show that expression of the flagellin and FliZ-dependent hemolysin genes is heterogeneous, differing between individual cells, and that a FliZ threshold controls the rate of transition between the OFF and ON states of FliZ-dependent gene expression at the single-cell scale. FliZ-modulated bimodal gene expression generated a mixed population of cells, with different levels of FliZ-dependent gene expression, resulting in the transient production of individuals with different potentials in terms of host interactions.

## Results

### FliZ is a Global Regulatory Protein Affecting the Transcription of Genes Encoding Flagellar and Non-flagellar Proteins

Our previous transcriptional analysis revealed that FliZ was required for the coupling of motility and hemolysin expression in *X. nematophila* and that FliZ bound directly to the promoter regions of the hemolysin and *flhDC* operons, functioning as an activator [13]. We investigated the whole FliZ regulon using RNA-Seq analysis to compare the gene expression profiles of the wild type strain F1 and the isogenic *fliZ* mutant at mid-exponential growth phase (OD_540_ = 0.5). More than 73 million Illumina sequences (36-base reads) were obtained for each sample. More than 82% of these sequences were of sufficiently high quality and could be mapped to at least one site in the *X. nematophila* F1 genome [30]. Transcriptomes were compared for each annotated feature between the wild type strain and the *fliZ* mutant (GEO accession number: GSE47365). We observed significant differences in expression between the *fliZ* mutant and the wild type strain for 278 coding sequences (|log_2_ fold change| ≥1; adjusted *P*-values**≤**0.05; Table S1), 235 of which were downregulated and 43 of which were upregulated in the *fliZ* mutant relative to the wild type strain. These genes were either isolated or clustered into 23 genomic regions scattered throughout the bacterial chromosome. The genes on which *fliZ* inactivation had the strongest effect are listed in Table 1.

**Table 1:** Genes differentially transcribed between the wild type F1 strain and the ΩfliZ mutant in exponentially growing cells, as a function of decreasing adjusted *P* value*.

			RNAseq		RT-qPCR
Label	Gene	Product	log2 fold change ratio (ΩfliZ/WT)	adjusted *p*-value (FDR)	log2 fold change ratio (ΩfliZ/WT)
***Genes positively regulated by FliZ***					
XCN3_2390004	*fliZ*	Flagellar transcriptional regulator	−8.47	0	
XCN3_870012	*xaxA*	Hemolysin component, XaxA	−6.44	0	−6.27
XCN3_870013	*xaxB*	Hemolysin component, XaxB	−6.36	0	
XCN3_2390006	*fliC*	Flagellin	−5.55	5E-296	−5.44
XCN3_2880003	*xhlA*	XhlA, Cell surface-associated hemolysin (TpsA)	−5.2	6E-275	−5.64
XCN3_2670009	*prtA*	Serralysin-like metalloprotease PrtA	−4.95	9E-254	−4.76
XCN3_1720008	*tse*	Methyl-accepting chemotaxis serine transducer	−4.52	1E-213	−4.44
XCN3_2410006	*flgK*	Flagellar hook-associated protein 1	−4.5	2E-213	
XCN3_2390007	*fliD*	Flagellar hook-associated protein 2	−4.26	8E-195	
XCN3_2410005	*flgL*	Flagellar hook-associated protein 3	−4.29	3E-194	
XCN3_2390005	*fliA*	RNA polymerase sigma factor for flagellar operon	−4.15	2E-184	−5.44
XCN3_1720007	*tas*	Methyl-accepting chemotaxis aspartate transducer	−4.09	3E-178	
XCN3_1720013	*motA*	Motility protein A	−4.08	9E-169	
XCN3_2410012	*flgF*	Flagellar basal-body rod protein FlgF	−3.77	1E-156	
XCN3_2410013	*flgE*	flagellar biosynthesis; hook protein	−3.74	3E-156	
XCN3_2410011	*flgG*	Flagellar basal-body rod protein FlgG	−3.75	4E-155	
XCN3_2410014	*flgD*	Basal-body rod modification protein FlgD	−3.74	2E-153	
XCN3_1720012	*motB*	Motility protein B	−3.76	4E-149	
XCN3_2390013	*fliF*	Flagellar M-ring protein	−3.69	2E-148	
XCN3_2410008	*flgI*	Flagellar P-ring protein	−3.68	1E-147	
XCN3_2410016	*flgB*	Flagellar basal body rod protein FlgB	−3.68	3E-147	−3.99
XCN3_1720005	*cheB*	Chemotaxis response regulator protein-glutamate methylesterase	−3.73	2E-141	
XCN3_2410010	*flgH*	Flagellar L-ring protein	−3.58	3E-139	
XCN3_1720011	*cheA*	Chemotaxis protein CheA	−3.52	1E-136	
XCN3_1720006	*cheR*	Chemotaxis protein methyltransferase	−3.8	3E-135	
XCN3_2410015	*flgC*	Flagellar basal-body rod protein FlgC	−3.5	6E-134	
XCN3_2390014	*fliG*	Flagellar motor switch protein FliG	−3.53	9E-133	
XCN3_2880004	*xhlB*	XhlB, XhlA hemolysin secretion/activation protein (TpsB)	−4.01	1E-131	
XCN3_740002	*xptA*	A component of insecticidal toxin complex (Tc)	−3.41	2E-129	−3.43
XCN3_1720004	*cheY*	Chemotaxis protein CheY	−3.55	6E-123	
***Genes negatively regulated by FliZ***					
XCN3_1640041	-	Conserved protein of unknown function	3.04	1E-22	
XCN3_1640042	-	Putative phage gene	2.74	2E-21	
XCN3_870020	-	Putative transcriptional regulator, TetR family	2.21	3E-20	1.97
XCN3_1790050	-	Conserved hypothetical protein	1.94	1E-19	2.18
XCN3_1090004	-	Conserved protein of unknown function	1.52	9E-16	
XCN3_1640043	-	Conserved protein of unknown function	2.09	8E-13	
XCN3_1090005	-	Conserved protein of unknown function	1.48	1E-12	
XCN3_1640047	-	Putative tail sheath protein	1.92	3E-12	
XCN3_1640044	-	Putative phage gene	2.15	8E-12	
XCN3_1090003	-	Conserved protein of unknown function	1.2	4E-11	
XCN3_1530004	*yfcH*	Epimerase family protein YfcH	1.3	7E-11	1.55
XCN3_2530030	*feoB*	Ferrous iron transport protein B	1.22	1E-10	1.22
XCN3_2410003	-	putative cysteine desulfurase (TRNA sulfur transferase), PLP-dependent	1.33	4E-10	
XCN3_2410002	-	Conserved protein of unknown function	1.36	4E-10	1.37
XCN3_1640046	-	Putative phage gene	1.9	5E-10	
XCN3_290001	*rpoS*	Sigma S factor of RNA polymerase, major sigma factor during stationary phase	1.15	5E-10	1.44
XCN3_1640045	-	Putative phage gene	1.96	7E-10	
XCN3_110011	*nilR*	NilR transcription factor	1.15	7E-10	
XCN3_110010	*nilQ*	NilQ	1.12	3E-09	1.27

* results obtained from DESeq analysis of the RNA-Seq-based comparison of the *Xenorhabdus nematophila* F1 strain and its *fliZ* isogenic mutant.

The protein-coding genes significantly downregulated in the *fliZ* mutant included all 47 flagellar protein-encoding genes, clustered in three flagellar regions (loci 13, 14 and 17; Table S1). Many other non-flagellar functional clusters were also upregulated by FliZ *(i)* the 14 genes (*xcnA–N*; locus 15) required for the synthesis of xenocoumacin, the major antimicrobial compound produced by *X. nematophila* [31]; *(ii)* the *pax* cluster (locus 10) encoding enzymes involved in synthesis of the Pax antimicrobial cyclolipopeptide [32], [33] and *(iii)* all 16 genes (locus 23) encoding putative components of a type VI secretion system [34], [35]. Non-flagellar genes directly regulated by FliZ, such as those encoding hemolysins (XaxAB and XhlAB) [13], were identified, as expected. However, more surprisingly, *xptA1*, encoding XptA1, the active component of a high-molecular weight protein toxin complex (Tc), was found to be strongly regulated by FliZ, and XptA2, a protein without insecticidal activity detected [36], was found to be weakly regulated by FliZ.

FliZ was initially described as a transcriptional activator, but we also identified individual genes and clusters of genes downregulated by this protein (Tables 1 and S1). Most of these downregulated genes are annotated as encoding hypothetical proteins of unknown function. However, FliZ also represses the transcription of a prophagic region (loci 11 and 12). The *rpoS* gene encoding the sigma factor σS was also found to be repressed by FliZ, consistent with the observed interference of FliZ with the expression of σS-dependent genes in *E. coli* [37]. We also found that FliZ downregulated the *nilQR* locus encoding NilR, a DNA-binding protein that, in turn, represses the transcription of *nilAB and nilC* required for colonization of the nematode host [38]. For validation of our differential RNA-Seq analysis, the expression level for 15 genes encoding factors potentially involved in the lifecycle of *Xenorhabdus* were also determined by real-time RT*-*PCR (Table 1). The fold-changes obtained with the two techniques were very similar, yielding a correlation coefficient (R^2^) of 0.96 (Table 1, Figure S1).

Taken together, these results demonstrate that FliZ is required for the efficient expression of the entire flagellar regulon in *Xenorhabdus*. FliZ also serves other functions, as a positive or negative regulator of the expression of numerous genes encoding non-flagellar proteins potentially involved in key steps of the *Xenorhabdus* lifecycle.

### FliZ Inactivation Leads to a Pleiotropic Phenotype in *Xenorhabdus*

Mutations in the *flhDC* and *fliAZ* operons of *X. nematophila* have been shown to affect swimming and swarming motilities, lipase and protease production, hemolysis and insect virulence [13], [20], [39]. However, the relative impacts of these regulatory factors on phenotypes remain unclear. We tried to elucidate the role of FliZ, by comparing the phenotypic characteristics of the wild type and of *flhD*, *fliAZ* and *fliZ* mutants with and without *fliZ* expression, using an inducible P_tet_-*fliZ* construct (Table 2). No difference was observed between strains in terms of antibiotic production, lecithin degradation and bromothymol blue adsorption. As expected [23], the *fliZ* mutant had no hemolytic activity on sheep blood agar and displayed an attenuated virulence phenotype in insects similar to that of the *fliAZ* and *flhD* mutants (Figure S2). However, the *fliZ* mutant was unable to hydrolyze Tween 20 and presented a weak ability to swim on motility agar plates; both these phenotypes are known to be FliA-mediated [20]. The ectopic expression of FliZ complemented all affected phenotypes in the *fliZ* mutant, but it restored only hemolytic activity in the *flhD* and *fliAZ* mutants (Table 2). The absence of complementation for lipase production and motility in these strains indicates that FliZ acts indirectly on these FliA-dependent phenotypes, through the positive feedback loop exerted by FliZ on *flhD* expression [13]. These data demonstrate that FliZ plays a key role, not only in hemolysin activity and full virulence in insects, but also in efficient motility and lipase activity in *Xenorhabdus*.

**Table 2:** Phenotypic characteristics of the *fliZ* mutant with and without complementation with a *fliZ*-expressing plasmid^a^.

		Btb adsorption^b^		Antibiotic production^c^		Lecithin degradation^d^		Sheep blood hemolysis^e^		Chemotaxis^f^		Lipolysis of Tween 20^g^	
Strain	Description	− aTc	+ aTc	− aTc	+ aTc	− aTc	+ aTc	− aTc	+ aTc	− aTc	+ aTc	− aTc	+ aTc
F1 (P*_tet_*-MCS)	Wild-type strain carrying empty vector	B	ND	+	ND	+	ND	T	ND	++	ND	+	ND
ΩfliZ (P*_tet_*-MCS)	*fliZ* mutant carrying empty vector	B	ND	+	ND	+	ND	−	ND	+	ND	−	ND
ΩfliZ (P*_tet_*-*fliZ*)	*fliZ* mutant carrying a P*_tet_*-*fliZ* construct	B	B	+	+	+	+	−	P	+	++	−	+
ΩfliA (P*_tet_*-*fliZ*)	*fliA* mutant carrying a P*_tet_*-*fliZ* construct	B	B	+	+	+	+	−	P	−	−	−	−
ΩflhD (P*_tet_*-*fliZ*)	*flhD* mutant carrying a P*_tet_*-*fliZ* construct	B	B	+	+	+	+	−	T	−	−	−	−

a . All plates were incubated for 2 days at 28°C before assay interpretation, unless otherwise indicated. aTc, anhydrotetracycline; ND, not determined.

b . Btb, bromothymol blue; B, dark blue colonies.

c . +, zone of *Micrococcus luteus* (laboratory collection) growth inhibition; median diameter = 35±6 mm.

d . +, halo up to 8 mm in diameter.

e . T, total hemolysis; P, partial hemolysis, −, no hemolysis on 24 h-old cultures.

f . Chemotaxis halo: ++, large spreading area (>20 mm); +, ∼10 mm; −, no spreading area.

g . We observed 72 h-old cultures on Tween plates. Qualitative evaluation of the halo surrounding the bacterial colony +, activity detected; −, no halo.

### The *Xenorhabdus fliZ* Mutant Is Impaired for Flagellin Production

**Figure 1:**
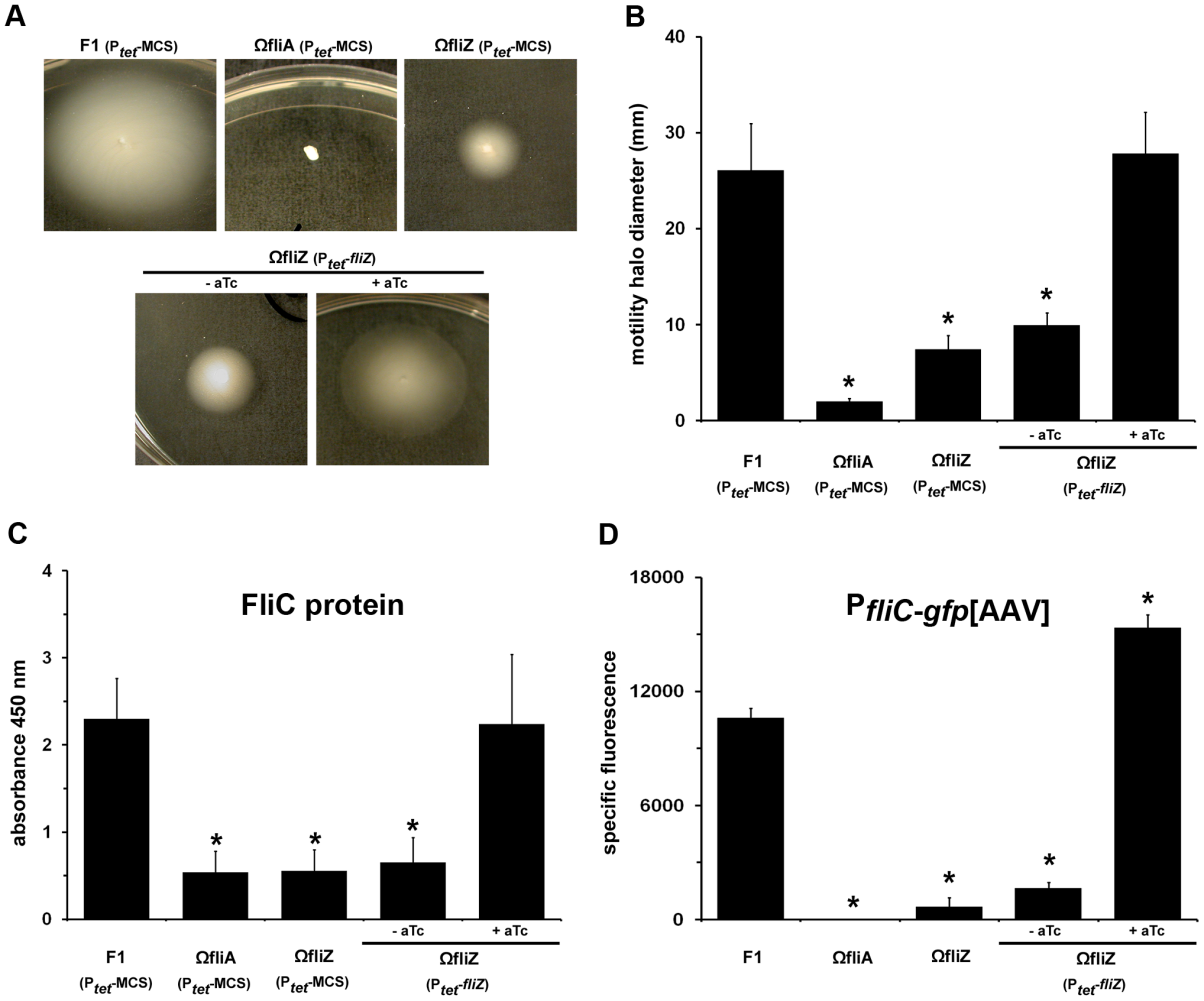
FliZ controls flagellin gene expression and motility phenotype in *Xenorhabdus*. Swimming motility assays were performed with wild type F1, *fliAZ* and *fliZ X. nematophila* strains carrying the empty vector P_tet_-MCS or a P_tet_-*fliZ* construct. Ectopic *fliZ* expression was induced by adding aTc (200 ng.ml^−1^) to the growth medium. Representative images of motility assays are shown in panel **A** and quantification of the motile phenotype (**B**) results from three independent assays. (**C**) The strains indicated were cultured in LB medium to late exponential growth phase and FliC protein was quantified in the bacterial extracts by immunodetection with anti-FliC antibodies. The data shown are the means and standard errors of four independent experiments. (**D**) Wild type F1, ΩfliA and ΩfliZ strains carrying the P*_fliC_*-*gfp*[AAV] construct or the ΩfliZ strain carrying the P*_fliC_*-*gfp*[AAV] - P_tet_-*fliZ* construct were grown to late exponential growth phase. Specific fluorescence is expressed as the ratio of GFP fluorescence to absorbance at 600 nm. The results shown are the means and standard errors of three independent assays. Significant differences from the wild type F1 strain (*p*-value<0.05, Student's *t*-test) are indicated by asterisks (*).

The *fliZ* mutants of *E. coli* and *S.* Typhimurium are fully motile and display only modest decreases in flagellar gene expression when grown in LB medium [15]. By contrast, the motility diameter developed by the *Xenorhabdus fliZ* mutant growing on LB motility agar plates was smaller than that obtained with the wild type strain, by a factor of 3.5 (Figure 1A and 1B). However, unlike the polar *fliAZ* mutation, *fliZ* deletion did not fully abolish the swimming capacity of *Xenorhabdus*. The *fliZ* (P_tet_-*fliZ*) strain recovered full motility in the presence of the inducer anhydrotetracycline (aTc) (Figure 1A and 1B). We then investigated whether the motility defect of the *fliZ* mutant resulted from lower levels of flagellin production. The amount of flagellin produced by the *fliZ* mutant, as estimated by ELISA, was smaller than that produced by the wild type, by a factor of 3.2, and was similar to the amount of FliC detected in the non motile *fliAZ* strain (Figure 1C). As expected, expression of the P*_tet_*-*fliZ* construct in the *fliZ* mutant fully restored flagellin production. We therefore used a P*_fliC_*-*gfp*[AAV] construct as a reporter for the level of expression of the flagellin gene. The GFP fluorescence signal was recorded in a bulk assay, on wild type, *fliAZ* and *fliZ* mutant cells grown for 15 h. As expected, GFP fluorescence was undetectable in the *fliAZ* mutant and reduced in the *fliZ* mutant by a factor 15 when compared to wild type (Figure 1D). Complementation assays, performed with a plasmid carrying both P*_fliC_*-*gfp*[AAV] and P*_tet_*-*fliZ* constructs, also resulted in high levels of GFP fluorescence if the *fliZ* strain was cultured in the presence of the aTc inducer. Together with the results of RNA-Seq experiments, these data demonstrate that the motility defect of the *fliZ* mutant results from a global decrease in the expression of flagellar genes, resulting in the production of low amounts of the flagellin monomer.

### The Expression of FliZ-Dependent Hemolysin and Class II–III Flagellar Genes Is Heterogeneous at the Single-Cell Level

**Figure 2:**
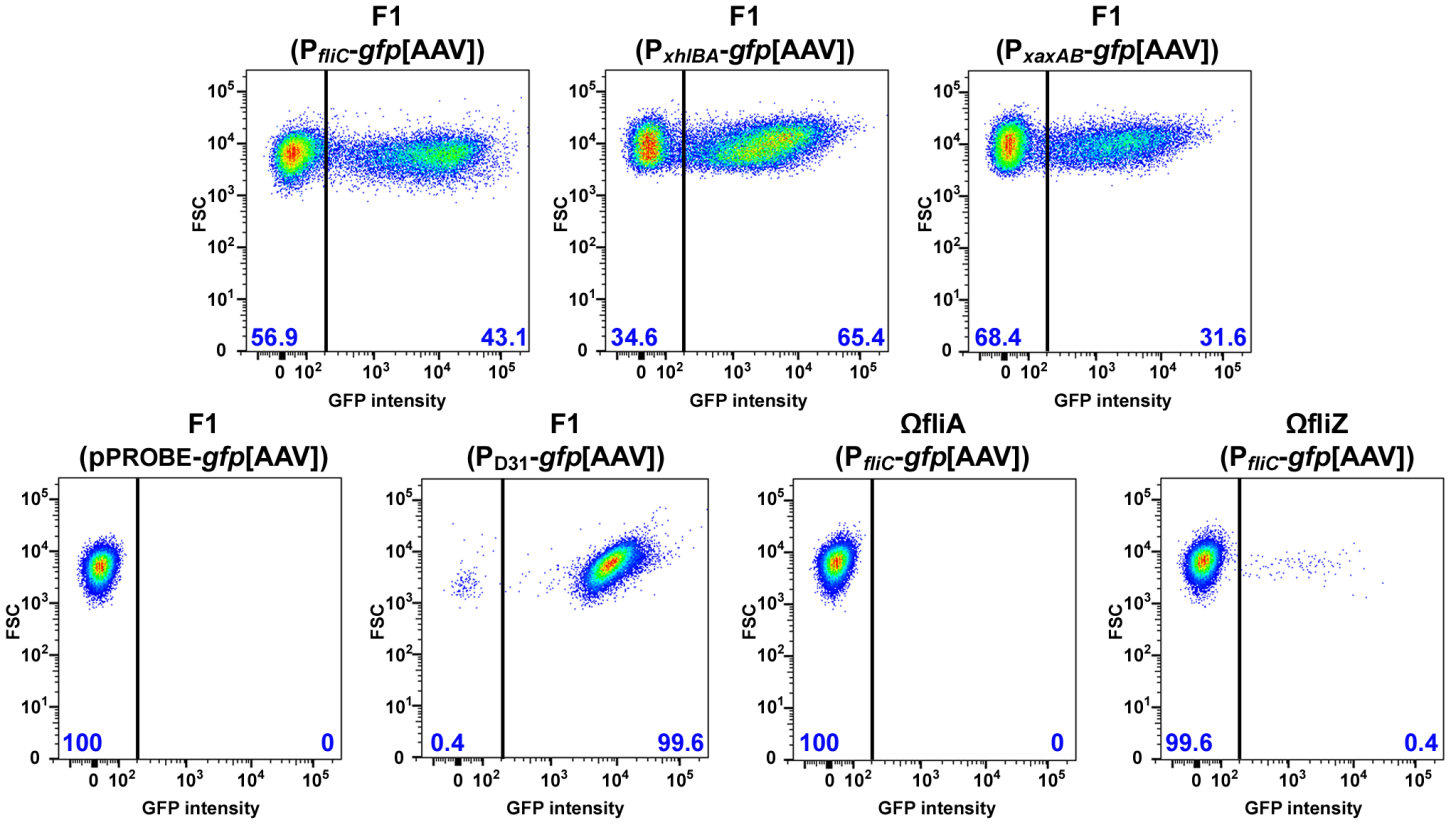
Bimodal expression of flagellin and hemolysin genes in *Xenorhabdus*. The strains indicated were grown in LB medium to mid-exponential growth phase (strains with P*_fliC_*-*gfp*[AAV], pPROBE-*gfp*[AAV] constructs or P_D31_-*gfp*[AAV]constructs) or mid-stationary growth phase (strains with P*_xaxAB_*-*gfp*[AAV] or P*_xhlBA_*-*gfp*[AAV] constructs) and GFP fluorescence signals were quantified in individual bacterial cells by flow cytometry. The pPROBE-*gfp*[AAV] vector carrying a promoter-less *gfp* gene and the P_D31_-*gfp*[AAV] construct carrying the *gfp* gene under the control of a constitutive promoter were used as negative and positive controls, respectively. Data are shown on two-dimensional dot plots, with the GFP signal on the x-axis and the forward scatter parameter (FSC) on the y-axis. Gates corresponding to GFP-negative and GFP-positive populations are shown and the corresponding percentages are indicated at the bottom left and bottom right of each image, respectively.

Microscopic observations performed during insect infection have revealed that the expression of the *fliC* and *xaxAB* genes in *Xenorhabdus* is heterogeneous differing between individual cells [23]. We investigated the heterogeneity of *fliC*, *xaxAB* and *xhlBA* gene expression during bacterial growth *in vitro*, by measuring the expression of these genes in the wild type strain carrying the P*_fliC_*-*gfp*[AAV], P*_xaxAB_*-*gfp*[AAV] or P*_xhlBA_*-*gfp*[AAV] fusion by flow cytometry. Two distinct populations (OFF [GFP-negative cells] and ON [GFP-positive cells]) were visualized, providing evidence of a bimodal distribution of cells, in terms of the expression of flagellin and hemolysin genes, in *Xenorhabdus* (Figure 2 and S3).

The *fliZ* mutant of *Xenorhabdus* is less motile and has lower levels of flagellar protein than the wild type. It is therefore possible that the motility phenotype is supported by the expression of the flagellar regulon by only a small number of cells. We tested this hypothesis, by measuring the expression of the P*_fliC_*-*gfp*[AAV] fusion gene in the *fliZ* and *fliAZ* mutant strains, at single-cell resolution. We detected no fluorescent bacteria for the *fliAZ* mutant or the wild type strain carrying a promoter-less *gfp* gene used as a negative control (Figure 2). By contrast, GFP-positive cells were clearly detected for the *fliZ* mutant carrying P*_fliC_*-*gfp*[AAV], albeit in much smaller numbers than for the wild type strain, corresponding to only 0.4% of all bacteria (Figure 2). These data suggest that the low levels of *fliC* gene expression observed in the *fliZ* mutant at the whole population level (Figure 1) result from a substantial decrease in the number of bacteria expressing the flagellin gene.

**Figure 3:**
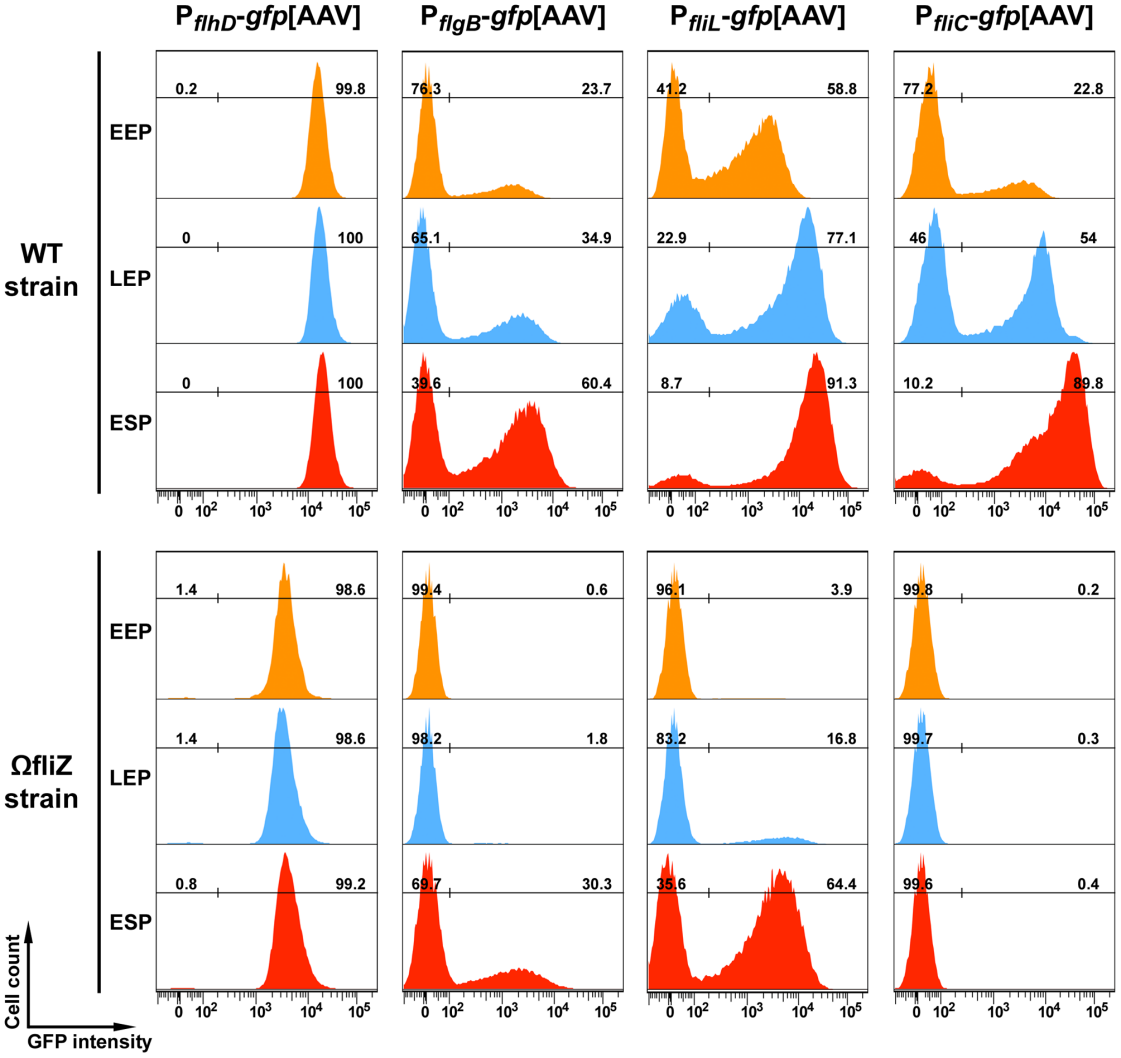
Time-course of P*_flhD_*, P*_flgB_* , P*_fliL_* and P*_fliC_* promoter activities in wild type and *fliZ* strains. During the growth of the indicated strains in LB medium, aliquots of the culture were collected during early exponential growth phase (EEP; OD_540_ ∼0.5), late exponential growth phase (LEP; OD_540_ ∼1.3) and early stationary phase (ESP; OD_540_ ∼2.5), and GFP fluorescence was analyzed in individual bacteria by flow cytometry. Data are represented as histograms, with GFP signal on the x-axis and cell number on the y-axis. Gates corresponding to GFP-negative and GFP-positive populations are shown and the corresponding percentages are indicated.

In *S.* Typhimurium, FliZ induces a switch in the kinetics of class II flagellar gene expression [28]. We investigated whether flagellar genes from classes I, II and III were expressed with a bimodal distribution in *Xenorhabdus*, by monitoring the expression of *flhD* (class I), *flgB* and *fliL* (class II) and *fliC* (class III) during time-course study of the growth of the wild type strain. As for *fliC*, the expression of the class II genes, *flgB* and *fliL*, was bimodal throughout the growth of the bacteria (Figure 3). The percentages of bacteria corresponding to the OFF and ON populations for the *fliL* and *fliC* genes fluctuated strongly over time (Figure 3), with the ON population accounting for about 90% of all bacteria when the culture reached the early stationary phase. By contrast, only a single ON population was observed for the master operon *flhDC*, regardless of the growth stage considered (Figure 3). Thus, heterogeneous gene expression is intrinsic to the flagellar cascade and not due to external factors affecting *flhDC* expression.

We evaluated the role of the FliZ positive feedback loop in the observed bimodal pattern of expression, by quantifying flagellar gene expression in the *fliZ* mutant, in which the FliZ feedback loop is inactivated. As previously observed, the percentage of *fliC*-expressing *fliZ* mutant bacteria did not exceed 1%, regardless of the growth stage considered. By contrast, the class II genes, *flgB* and *fliL*, were expressed with a bimodal distribution in both the wild type and *fliZ* mutant strains. However, GFP-positive populations emerged later in the *fliZ* mutant strain. The expression of *flhD* remained unimodal in the *fliZ* mutant cells, but was much weaker (by a factor of 5) than that in the wild type strain (Figure 3). The FliZ feedback loop is, therefore, necessary for the early dynamics of class II flagellar gene expression, but dispensable for the heterogeneous expression of flagellar genes in *X. nematophila*.

### Intracellular FliZ Levels in *Xenorhabdus* Determine FliZ-Dependent Gene Activation at the Single-Cell Scale

**Figure 4:**
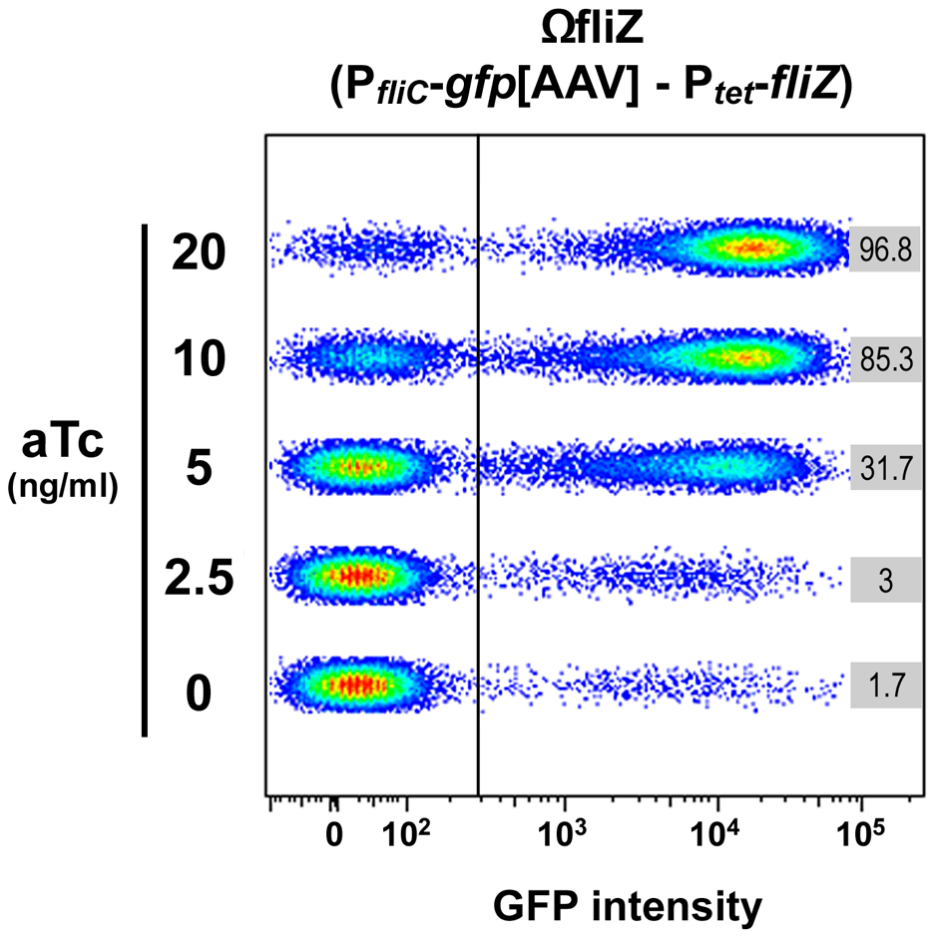
FliZ protein level governs the transition between OFF and ON states of *fliC* gene expression in individual *Xenorhabdus* bacteria. The *fliZ* strain carrying the P*_fliC_*-*gfp*[AAV] - P_tet_-*fliZ* construct was grown in LB and the final concentration of aTc indicated was added when the OD_540_ reached 0.1. Three hours after aTc addition, bacteria were collected and GFP fluorescence signal was recorded in individual cells by flow cytometry. Data are shown on graphs consisting of five two-dimensional dot plots with the GFP signal on the x-axis and the forward scatter parameter (FSC) on the y-axis. Gates corresponding to GFP-negative and GFP-positive populations are indicated and the percentages of GFP-positive cells are indicated on the right, for each sample.

The FliZ feedback loop is not directly involved in the generation of bimodal expression patterns for class II flagellar genes, but FliZ may exert its activity through positive control over flagellar gene expression, mediating the transition between OFF and ON populations. We tested this hypothesis, by carrying out complementation assays with the plasmid carrying both P*_fliC_*-*gfp*[AAV] and P*_tet_*-*fliZ* constructs. The addition of aTc led to a dose-dependent increase in the proportion of cells belonging to the ON population, which reached more than 96% for the highest concentrations of the inducer (Figure 4). However, the pattern of gene expression remains bimodal overtime and more specifically for the intermediate concentration of aTc (5 to 10 ng/ml). A slight increase in the amount of aTc, from 2.5 to 5 ng/ml, induced a shift of one third of the cells into the ON state. These results clearly demonstrate rheostatic control, by FliZ, of the rate of transition between OFF and ON states of flagellin gene expression at the individual scale. In contrast, the complementation assays of the *flhD* mutant with the plasmid carrying both P*_tet_*-*flhDC* and P*_fliC_*-*gfp*[AAV] causes a more homogeneous response in cell population where almost all cells are either ON or OFF (Figure S4).

**Figure 5:**
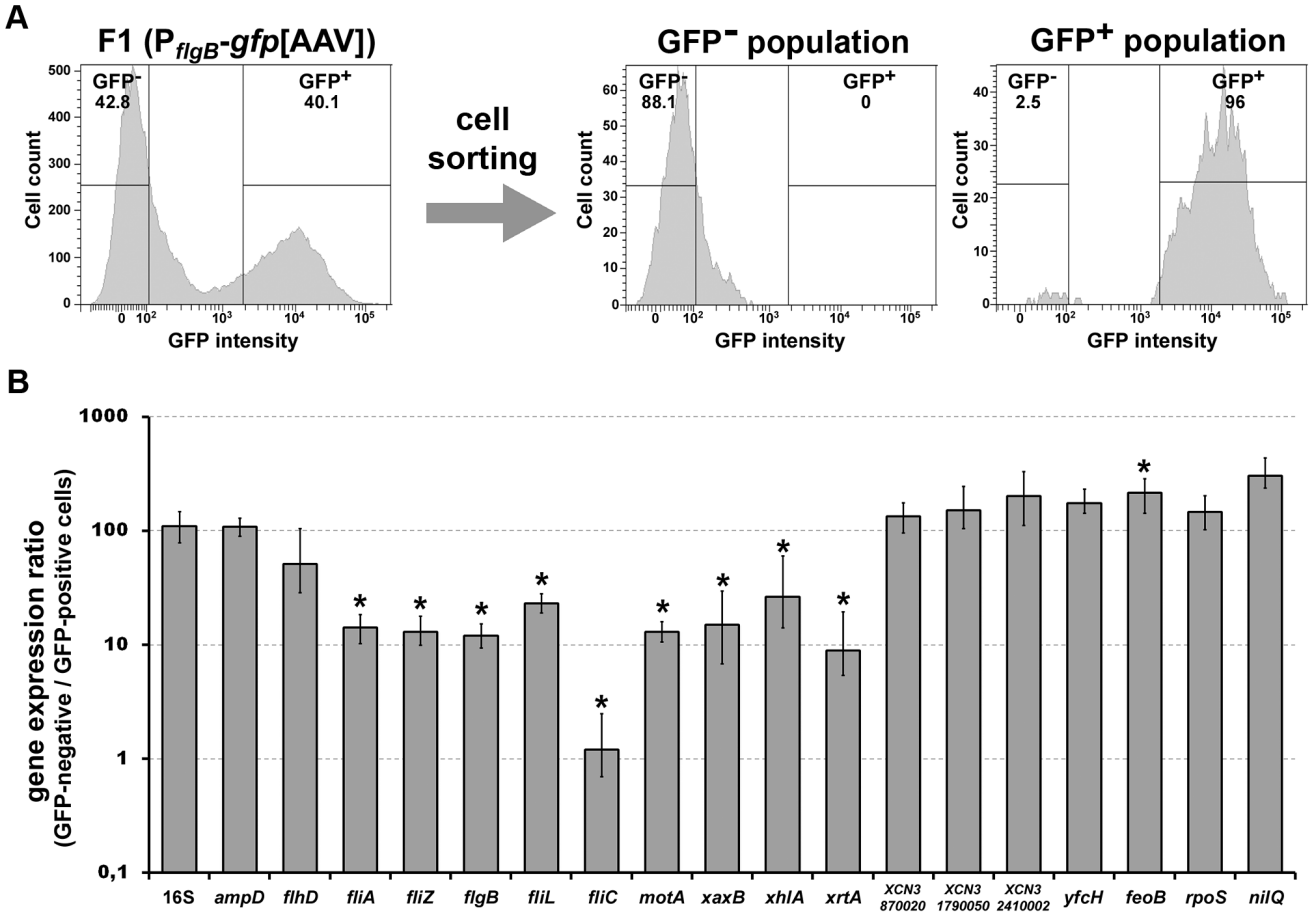
Single-cell analysis of the expression of FliZ-dependent genes. (**A**) The wild type F1 strain carrying the P*_flgB_*-*gfp*[AAV] construct was grown in LB medium to early-exponential growth phase (EEP) and GFP-negative and GFP-positive populations were then sorted by FACS. The GFP fluorescence signal was quantified in individual bacterial cells before (left panel) and after (right panels) cell sorting. Gates corresponding to GFP-negative and GFP-positive populations are shown and the corresponding percentages are indicated. Approximately 30,000 and 3,000 cells were analyzed for unsorted and sorted samples, respectively. (**B**) Total RNA was extracted from GFP-negative and GFP-positive sorted cells and used for RT-qPCR analysis with internal primers specific for the indicated genes. mRNA levels were normalized against those of a reference gene (*recA*). Data are presented as a ratio of values for GFP-negative and GFP-positive samples. A ratio of 100 indicates no difference in expression level between GFP-negative and GFP-positive cell samples. Significant differences (*p*-value<0.05) are indicated by asterisks (*).

These findings also suggest that the FliZ-modulated dynamic heterogeneity in flagellar gene expression may give rise to an “OFF” state in which the FliZ regulon is switched off, and an “ON” state in which FliZ-dependent genes are expressed in a controlled manner. For the validation of this hypothesis, we separately quantified the levels of FliZ-dependent gene transcripts in ON and OFF populations, after cell sorting. The wild type strain carrying the P*_flgB_*-gfp[AAV] fusion displayed a constant bimodal pattern of expression, regardless of the growth phase considered (see Figure 3). We therefore used this strain to separate GFP-negative and GFP-positive bacterial cells by fluorescence-activated cell sorting (Figure 5A). As expected, the sorting of bacteria in the early exponential growth phase (EEP) showed *flgB* transcript levels to be eight times higher in the GFP-expressing population than in the GFP-negative population. A slight decrease in *flhD* transcription was found to be associated with significant decreases in the levels of expression of all the class II and class III flagellar genes studied, by factors of 4 to 80 (for *fliC*) in non fluorescent cells with respect to GFP-expressing cells. Non-flagellar genes encoding hemolysins (XaxAB and XhlBA) or protease PrtA/XrtA, which were previously shown to be upregulated by FliZ in our RNA-Seq analysis (Table 1), also displayed significant downregulation in GFP-negative bacterial cells. Only one gene downregulated by FliZ, *feoB*, displayed significantly higher levels of expression in non fluorescent than in fluorescent cells. However, the seven FliZ-repressed genes, including the genes encoding the regulators RpoS and NilQ, were only slightly more expressed in the OFF state (Figure 5B). Overall, these data indicate that the two cellular states resulting from bimodal expression of the flagellar cascade express the FliZ regulon differentially. They also suggest that the amount of FliZ present within the cell governs its fate.

## Discussion

**Figure 6:**
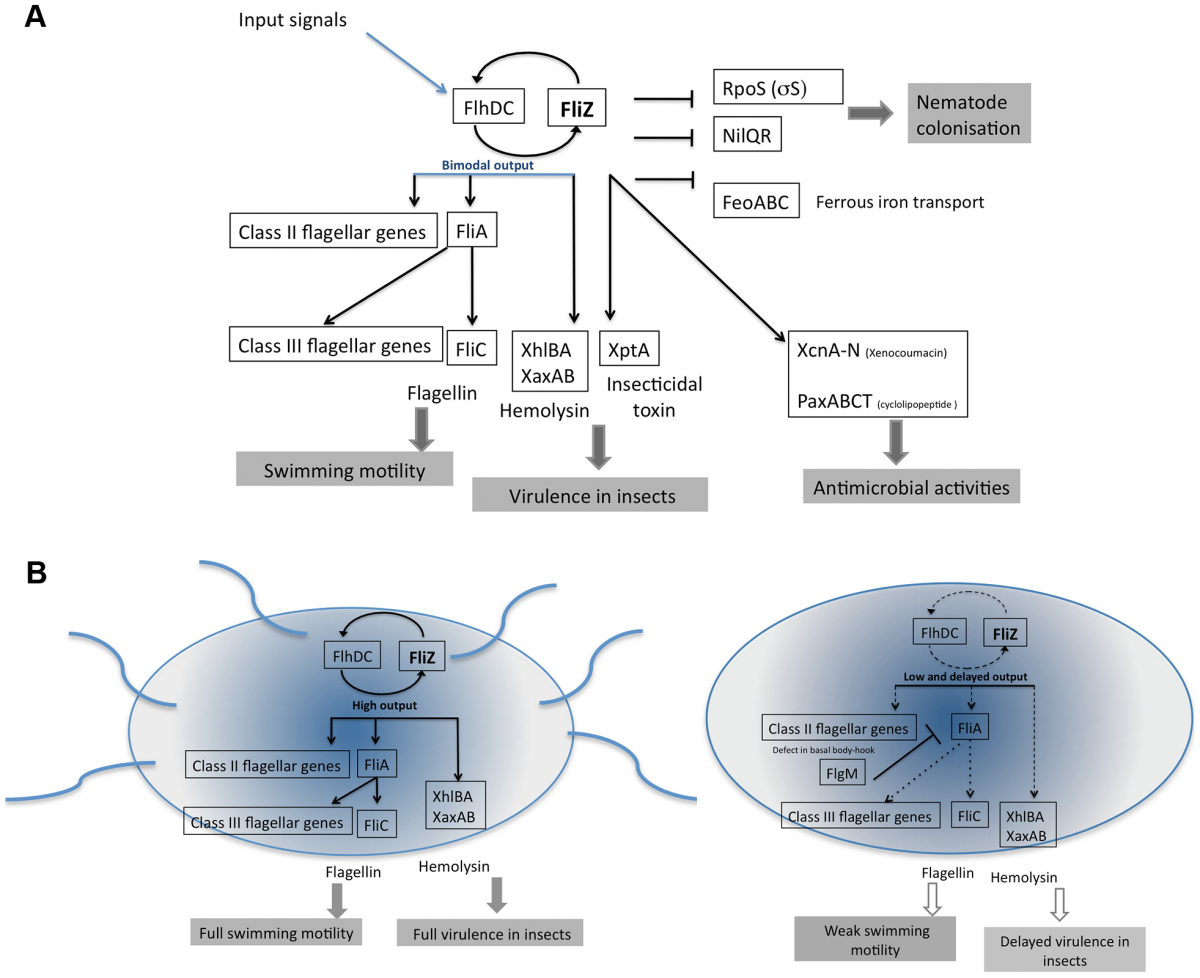
Model summarizing the FliZ regulon and FliZ-modulated cell heterogeneity in *X. nematophila*. (**A**). At the top of the network, FliZ coordinates the expression of flagellum-driven motility through a feedback effect on the expression of *flhD* (the flagellar regulon) and genes encoding non-flagellar proteins involved in the lifecycle of the bacterium and its interaction with invertebrates [19]. Numerous regulators (input signals) control the master operon *flhDC* of *Xenorhabdus* [19]. The resulting patterns of expression of class II–III flagellar genes (strictly FlhD-dependent) and hemolysin-encoding genes directly controlled by FliZ [13] are bimodal at the population level. In addition, FliZ directly or indirectly upregulates the expression of genes encoding insecticidal toxins and antimicrobial compounds and downregulates the expression of two genes encoding the transcriptional regulators σS (rpoS) and NilR, and a structural gene, *feoABC*. (**B**) The image shows the mixed bacterial population coexisting during the exponential growth of *X. nematophila*. On the left, a motile bacterium is shown. When the stochastic expression of the circuit generates large amounts of FliZ, this molecule exerts positive feedback on *flhDC* expression, upregulating the flagellar cascade and FliZ-dependent hemolysin genes. Consequently, the noisy expression of class II flagellar genes is reduced and cells fully express class II and III flagellar genes, resulting in the motility phenotype. On the right, a non-motile bacterium is shown. We suggest that the lower level of FlhD-FliZ output delays and desynchronizes class II gene expression, probably impairing completion of the basal body-hook structure. The FlgM protein, an anti-sigma-28 factor, binds FliA directly in *E. coli*, preventing class III promoter transcription until after hook-basal body completion [9]. Thus, the accumulation of FlgM in cells probably blocks the transcription of class III genes, including that encoding flagellin, resulting in a non motile state. The impact of the FliZ-mediated circuit on virulence is discussed in the text.

Despite increasing numbers of studies, we still know little about the precise role of FliZ in the regulation of flagellar gene expression and the coupling of flagellar regulation with the expression of virulence factors in pathogenic bacteria. This study provides comprehensive insight into the FliZ regulation circuit in individual cells of *Xenorhabdus nematophila*. RNA-Seq analysis revealed that FliZ was required for the efficient expression of all flagellar genes, through the positive feedback loop controlling the expression of *flhDC*, the master regulator of the flagellar cascade. FliZ was also found to up- or downregulate the expression of many genes encoding non-flagellar proteins potentially involved in key steps of the *Xenorhabdus* lifecycle (see the proposed model in Figure 6A). As already observed in the course of insect infection [23], we demonstrate here that the FliZ-dependent regulon, with the exception of the *flhDC* operon, is expressed in a bimodal manner in exponentially growing bacteria, leading to the establishment of subsets of cells in which FliZ-dependent genes are expressed at high or low levels (see Figure 6B). According to our model, FliZ controls the rate of transition between the “OFF” and “ON” states at the individual scale.

### FliZ Is Required for the Efficient Expression of All Flagellar Genes and for Full Motility

*X. nematophila* FliZ is a transcriptional regulator that binds *in vivo* to unspecified regions of DNA upstream from the *flhDC* master operon, thereby exerting positive feedback on *fliAZ* expression [13]. The RNA-Seq analysis described here demonstrated a positive impact of FliZ on the expression of all the genes belonging to the flagellar cascade. Gradual decreases in the expression levels of class I, II and III flagellar genes were observed in the *fliZ* mutant (Table S1). It is likely that the lower level of FlhD in absence of FliZ feedback decreases class II gene expression that in turn prevents late class III promoter transcription through the action of FlgM control (see legend of Figure 6B for more details). Here, we also showed that the effects of the FliZ positive feedback loop on *flhDC* expression are critical for efficient motility in *Xenorhabdus*. Moreover, ectopic *fliA* expression in the *fliZ* mutant did not restore the full motility of *X. nematophila* (G. Jubelin, unpublished data), highlighting the key role of the FliZ protein in flagellum-driven motility.

As mentioned above, there are several major differences between the flagellar regulation circuit of *X. nematophila* and those of *E. coli* and *S.* Typhimurium [13]. One of these differences concerns the regulatory elements in the *fliAZ* promoter region. Unlike the *fliAZ* operon in *Xenorhabdus*, which is controlled principally by FlhDC [13], the *fliAZ* operons of *E. coli* and *S.* Typhimurium have two promoters: a class II promoter, which is recognized by the sigma 70 RNA polymerase in the presence of the FlhD_4_C_2_ activator complex, and a class III promoter, which is recognized by the FliA/sigma 28 RNA polymerase [7], [40]. However, the role of the class III promoter remains a matter of debate, because a recent study showed that the class III *S.* Typhimurium *fliA* transcript was not significantly translated when FliZ was produced from both the class II and class III transcripts [41]. The role of FliZ in the regulation of the flagellar circuit also differs considerably between the various enterobacterial species studied. Indeed, FliZ upregulates motility in *S.* Typhimurium [15], whereas it slightly represses motility in *E. coli* [37]. Like that of *X. nematophila*, the FliZ of *E. coli* interferes with *flhDC* expression by binding to a sequence downstream from the transcriptional start site, which resembles the −10 element of a cryptic σS-dependent promoter [37]. However, this consensus sequence is not found in the promoter region of the *X. nematophila flhD* gene. In *S.* Typhimurium, FliZ activates flagellar gene expression indirectly, by binding to the promoter region of the *nlpC* operon, repressing the transcription of this operon, which also controls expression of the *ydiV* gene encoding an anti-FlhDC factor active in minimal media [15]. However, no *ydiV* homologs are present in the *X. nematophila* genome. FliZ may exert direct or indirect feedback on *flhDC* expression in many bacterial species, but its overall impact on motility behavior and the expression of FliZ-targeted genes differs considerably between motile bacterial species. This led us to examine the primary function of FliZ in the flagellar cascade network more closely and to suggest that changes to the FliZ regulation circuit may play a key role in evolution, allowing motile bacterial species to adapt to their specific ecological niches.

### FliZ-Modulated Bimodality and Control of Flagellar Gene Expression

Single-cell analysis revealed unexpected heterogeneity in gene expression in clonal bacterial populations in which particular gene circuits were either ON or OFF in individuals. Population heterogeneity has been reported for various phenotypes, including competence for sporulation, DNA uptake, biofilm formation and persistence in the presence of antibiotic treatment (see [42] for a review). Heterogeneity has also been reported for *B. subtilis*, in which a motile state and a sessile state coexist in growing bacterial populations [29]. Moreover, the use of tools favoring enrichment in noisy promoters in *S.* Typhimurium has revealed that two promoter sequences regulating genes involved in flagellum synthesis, *fliC* and *flgK*, display the highest levels of variation [27]. Noise can be exploited under certain conditions, to generate phenotypic heterogeneity. In the presence of positive regulatory feedback, a graded expression can be converted to a binary response, in which cells express a certain gene at high or low levels [42]. At the population level, this switch-like behavior may result in a bimodal distribution of gene expression, with the stable propagation of these differences to daughter cells. This type of gene expression pattern is commonly referred to as bistability [42]. We found that the expression patterns of class II and III flagellar genes were bimodal in *X. nematophila*. However the phenomenon we observed in *X. nematophila* is not a true bistable mechanism as the bimodality is transient for the expression of some flagellar genes (Figure 3). Surprisingly, single-cell analysis revealed differences in the expression dynamics of two class II genes, both of which were strictly FlhD-dependent. Expression of the *flgB-L* operon remained bimodal over time, whereas the *fliL-Q* promoter was switched on in almost all cells in early stationary phase (Figure 3). As the transition times are probably random, noisy expression at the population level probably results in cells expressing class II genes at different times. By contrast, the expression pattern of the master operon *flhDC*, which is controlled by numerous regulators [19], remained unimodal over time. Model-based predictions suggest that ‘democratic’ networks, in which a large number of genes mutually regulate each other, might limit variation in information flow by facilitating the emergence of a consensus decision. By contrast, ‘autocratic’ subnetworks may permit variation in information flow, by allowing expression levels within key regulatory hubs to differ between individuals in a population, resulting in different phenotypes [43]. Because the single class I operon, comprising the *flhDC* genes, is the master operon of the flagellar transcriptional hierarchy, these predictions may explain the noisy expression of the class II flagellar genes.

In the absence of *X. nematophila* FliZ, class II gene expression is induced later than in wild type cells and follows a bimodal pattern (Figure 3). These results also show that FliZ does not primarily induce heterogeneity in the expression of the class II flagellar genes in *X. nematophila*. A similar transient heterogeneity in class II flagellar gene expression has been reported for wild type cells of *S.* Typhimurium. By contrast to our observations in *Xenorhabdus*, *fliZ* mutation in *S.* Typhimurium causes a homogeneous response in the individual cells of the population [28]. The authors of this previous study suggested that the autogeneous FliA positive feedback loop resulted in heterogeneous expression from class II and III promoters suggesting an important role of FliZ in regulating flagellum assembly. As *X. nematophila* FliA has no effect on transcription of the *fliAZ* operon [13], this model cannot be applied to *X. nematophila*. Instead, we propose a model for *X. nematophila* in which the amount of FliZ within the cell has a major effect, fine-tuning the dynamics of the bimodality of flagellar gene expression. This model is based on the response of a cell population to a gradient of FliZ and shows that FliZ exerts rheostatic control over the rate of transition between the OFF and ON states of flagellin production (Figure 4). In the presence of FliZ-mediated positive feedback (Figure 6B), the noisy expression of class II flagellar genes is rapidly converted to a transient binary response, in which most of the cells strongly express class II and III flagellar genes, resulting in a motility phenotype at the population level. In the *fliZ* mutant, the weaker input of FlhD delays class II gene expression, probably resulting in a smaller number of cells concomitantly expressing class II flagellar genes. The heterogeneity of cellular dynamics and lower output would be expected to impair completion of the basal body-hook structure, leading in turn to the downregulation of class III gene expression through FlgM, resulting in a smaller number of motile cells (Figure 6B). This might explain why about 50% of the cells expressed class II genes whereas less than 1% of the cells of the *fliZ* mutant expressed the *fliC* gene. This scenario would account for the weak flagellum-driven motility of the *fliZ* mutant, due to the expression of class III genes by a much smaller proportion of the bacteria in the population.

### Impact of FliZ-Modulated Bimodality on Bacterial Phenotypes and Interactions with the Host

In addition to its role in flagellar regulation, FliZ has been shown to regulate the expression of a number of non-flagellar genes, either directly or indirectly, in *X. nematophila* [13] and other enteric bacteria. These genes include the pathogenicity island 1 (SPI1) genes [16], [44], [45] and type 1 fimbrial genes [46] in *S.* Typhimurium. Our RNA-Seq analysis revealed that FliZ up- or downregulated the expression of genes encoding many non-flagellar proteins potentially involved in key steps of the *Xenorhabdus* lifecycle. As expected, FliZ was found to upregulate hemolysin gene expression by binding directly to the *xaxAB* and *xhlAB* promoter regions [13]. Through positive feedback on *fliAZ* expression, FliZ also modulates the expression of the FliA-dependent gene *prtA* (also called *xrtA*), which encodes a protease [20], [47]. The expression of the FliA-dependent lipase gene, *xlpA* [20], [48], was not FliZ-dependent in our RNA-Seq analysis of exponentially growing bacteria, but significant differences were observed in a stationary phase assay (A. Lanois, unpublished data). We have yet to identify the bacterial factors potentially accounting for the delayed virulence pattern observed with the *fliZ* (Figure S2) and *flhD* mutants [39]. We previously showed that the FliZ-dependent hemolysin XaxAB, which strongly induces necrosis and apoptosis in insect immunocompetent cells [21], was not required for full virulence. One interesting candidate is XptA, a high-molecular weight toxin complex (Tc) protein with insecticidal effects found in *Xenorhabdus* and *Photorhabdus* [49]. Indeed, the Tc makes a major contribution to *Xenorhabdus* virulence, as demonstrated by the virulence defect of an *xptD1* mutant [50]. Thus, the loss of expression of *xptA* in the *fliZ* mutant, as revealed by our RNAseq data probably explains its attenuated virulence phenotype.

The repressive effects of FliZ are weak in *X. nematophila*. The genes annotated as FliZ-repressed include two genes encoding regulators, RpoS, the sigma factor σS, and NilR, which is indirectly involved in the colonization of the nematode host by *Xenorhabdus* [38], [51]. The *rpoS* mutation in *X. nematophila* is also associated with enhanced flagellum-driven motility [38], [51]. In *E. coli*, FliZ plays a key role in determining cell lifestyle: the FlhDC-controlled flagellum-based motility or a σS-dependent adhesive-sedentary lifestyle [37], [52]. The transition between the motile state of *Xenorhabdus* in insects and its mutualistic state, in which it adheres to the intestinal region of a soil-dwelling nematode, may also be regulated by interplay between RpoS and FliZ. However, the RpoS-dependent regulon has yet to be deciphered in *X. nematophila*.

Finally, we showed, by a combination of fluorescence-activated cell sorting and RT-qPCR quantification, that the two subpopulations coexisting during growth *in vitro* display differential expression of almost all the messenger RNA markers of the FliZ regulon. *In vivo* real-time expression analysis with an unstable GFP monitoring system has shown that FliZ target genes are upregulated just before the death of the insect, with expression levels peaking, at population level, in the larval cadavers. In addition, microscopic observations of insect cadavers have shown that FliZ-dependent gene expression in *Xenorhabdus* is heterogeneous, with differences observed between individuals [23]. FliZ-modulated bimodal expression may, therefore, lead to the generation of several subtypes of cells with different virulence potentials within an isogenic population of infecting bacteria (Figure 6B). It remains unclear why *Xenorhabdus* generates a mixed population of cells, some of which produce flagella, whereas others do not. Bacterial flagellins elicit innate immune responses in mammals and plants [1]. However, to our knowledge, no flagellin receptor has been described in insects, and it therefore remains unclear why variation in the expression of flagellar genes might be advantageous for insect infection. In the closely related association between *Photorhabdus* and its nematode host, the inversion of a single promoter, mediated by a site-specific recombination event, allows the bacteria to switch from a pathogenic to a mutualistic state [53]. Further studies are therefore required, to provide a clear demonstration that the bimodal expression of virulence factors constitutes a strategy for generating specialized cell types capable of surviving in different invertebrate niches, in *X. nematophila*.

## Materials and Methods

### Bacterial Strains, Plasmids and Growth Conditions

The strains and plasmids used in this study are listed in Table S2. Bacteria were grown routinely in Luria-Bertani (LB) medium or Mot broth (1% tryptone, 0.5% NaCl, 10 mM MgSO_4_) at 28°C (*X. nematophila*) or 37°C (*E. coli*). For motility assays, agar plates were prepared with LB broth supplemented with 0.35% agar. Antibiotic production, bromothymol blue adsorption, lecithinase, lipolytic and hemolytic activities were assessed as previously described [54]. When required, antibiotics were used at the following final concentrations: kanamycin, 20 mg.l^−1^, gentamicin, 30 mg.l^−1^ and chloramphenicol, 20 mg.l^−1^ for *E. coli* strains and 15 mg.l^−1^ for *X. nematophila*. P*_tet_* constructs were induced by adding anhydrotetracycline (aTc) at a final concentration of 0.2 ng.l^−1^, unless otherwise indicated.

### Construction of the *fliZ* Mutant

The regions upstream and downstream from *fliZ* (partial *fliA* and *putA* genes, respectively) were amplified by PCR with the fliA-Xba-f and fliA-BamHI-r primers for the upstream region and the putA-BamHI-f and putA-XhoI-r primers for the downstream region. The two 700 bp fragments obtained were inserted, together with the omega interposon cassette from pHP45-ΩCm conferring resistance to chloramphenicol, into pJQ200KS, to introduce the ΩCam cassette between the two PCR fragments. The resulting plasmid, pGJ906, was then used to transform *E. coli* strain S17.1 and was introduced into *X. nematophila* F1 in a mating experiment. Cm^r^ and Suc^r^ exconjugants were selected on LB agar supplemented with 4% sucrose and chloramphenicol. Omega insertion was confirmed by PCR analysis and the loss of hemolytic activity of the resulting *fliZ* strain was checked on sheep blood agar plates.

### Molecular Genetic Techniques and RNA Preparation

DNA manipulations were carried out as previously described [55]. Plasmids were introduced into *E. coli* by transformation and transferred to *X. nematophila* by conjugative mating [39]. All constructs were sequenced by Millegen (Labège, France). The primers used in this study (Eurogentec) are described in Table S3.

Total RNA was extracted with the RNeasy Protect Bacteria miniprep (for RNA-Seq experiment) or the RNeasy Micro kit (for sorted cells, from Qiagen) including DNase I incubation in accordance with the manufacturer's recommendations. For each RNA preparation, we assessed DNA contamination by carrying out a control PCR. The quantity and quality of total and messenger RNA, respectively, were assessed with a NanoDrop 2000 spectrophotometer (Thermo Scientific) and an Agilent 2100 Bioanalyzer with the RNA 6000 Nano LabChip kit (Agilent).

Material for RNA-Seq analysis was prepared by extracting total RNA from the *Xenorhabdus* wild type strain and the *fliZ* mutant grown in Mot broth (OD_540_ = 0.5) (six independent biological replicates per strain) and pooling equal amounts of total RNA from three replicates of the same strain together, to generate two biological samples for each strain, which were subjected to two successive rounds of ribosomal RNA depletion with the Microbe Express kit (Ambion) according to the manufacturer's instructions.

### RNA Sequencing

RNA-Seq libraries were constructed with the Truseq RNA sample preparation kit from Illumina. Briefly, for each sample, 100 ng of rRNA-depleted RNA was chemically fragmented. The first cDNA strand was generated by reverse transcription with random hexamer primers and SuperScript II Reverse Transcriptase (Life Technologies) and the second strand was then synthesized. A blunt-ended double-stranded DNA was then generated by repair techniques. A single “A” nucleotide was added to the 3′ end and ligation was carried out with Illumina's indexed adapters. After 15 cycles of PCR, libraries were validated with a DNA 1000 Labchip on a Bioanalyzer (Agilent) and quantified with a KAPA qPCR kit. For each sequencing lane, two libraries were pooled in equal proportions, denatured with NaOH and diluted to 8 pM before clustering. Clustering and 50 nt single-read sequencing were performed according to the manufacturer's instructions. Image analysis and base-calling were carried out with HiSeq Control Software (HCS 1.5.15) and a RTA component (RTA 1.13.48). Finally, demultiplexing was carried out with CASAVA (1.8.1).

### RNA-Seq Analysis

Transcriptomic high-throughput sequencing data were analyzed with a bioinformatic pipeline implemented within the Microscope platform [56]. The pipeline currently used is a “Master” shell script that launches the various parts of the analysis (i.e. a collection of Shell/Perl/R scripts) and checks that all tasks are completed without error. We first assessed RNA-Seq data quality by including options, such as read-trimming or the use of merging/split paired-end reads. We then mapped reads onto the contigs of the *X. nematophila* F1 genome sequence (accession number: CAVM000000000) with the SSAHA2 package [57], which combines the SSAHA searching algorithm (sequence information encoded in a perfect hash function) for identifying regions of high similarity, and the cross-match sequence alignment program, which aligns these regions, using a banded Smith-Waterman-Gotoh algorithm. An alignment score covering at least half of the read is required for a hit to be retained. We minimized the false positive discovery rate, by using SAMtools (v.0.1.8) to extract reliable alignments from SAM-formatted files. The number of reads matching each genomic object harbored by the reference genome was then calculated with the Bioconductor-Genomic Features package. If reads matched several genomic objects, the count number was weighted so as to keep the total number of reads constant. Finally, the Bioconductor-DESeq package [58] was used with default parameters for the analysis of raw count data and to determine whether expression levels differed between conditions. The complete dataset from this study has been deposited in the GEO database under accession no. GSE47365.

### RT-qPCR Analysis

RT-qPCR was performed in two steps. First, the cDNA was synthesized from 1 µg of total RNA from each replicate used for RNA-Seq (0.2 µg of total RNA was used for sorted cells), with Super Script II Reverse Transcriptase from Invitrogen and random hexamers (100 ng.µl^−1^) from Applied Biosystems. We then carried out qPCR in triplicate with the LightCycler 480 SYBR Green I Master kit from Roche Diagnostics, with 1 µl of cDNA synthesis mixture (diluted 1∶100 or 1∶20) and 1 µM of specific primers for the genes studied (Table S3). The enzyme was activated by heating for 10 minutes at 95°C. All qPCRs were performed in three technical replicates, with 45 cycles of 95°C for 5 s, 60°C for 5 s and 72°C for 10 s, and were monitored with the Light Cycler 480 system (Roche). Melting curves were analyzed for each reaction and each curve contained a single peak. The *recA* gene was used as the reference housekeeping gene and 16S, *mre* or *ampD* was used as an internal control. The data for each sample are expressed relative to the expression level of *recA*, as follows [59]: 
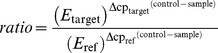
. The relative expression ratio for a target gene was calculated on the basis of its real-time PCR efficiency (E) and the crossing point (CP) difference (Δ) between a sample and the control (ΔCP_control – sample_). Crossing points (CP) for each sample and each gene were calculated with LightCycler480 (Roche) software, using second-derivative maximum analysis and the CP medians of the technical replicates for each biological sample used. All relative quantifications were assessed with REST software 2009, using the pairwise fixed randomization test with 2,000 permutations, with PCR efficiencies calculated with serial dilutions of a mixture of cDNAs [59]. This method provided a relative quantification of the expression of a target gene with respect to a reference gene, for the comparison of the wild type and *fliZ* mutant strains or of GFP-positive and GFP-negative cells.

### Construction of Plasmids Expressing *gfp*[AAV] under the Control of *fliC*, *flhD*, *flgB* or *fliL* Gene Promoters and of Plasmids Expressing *fliZ* or *flhDC* under the Control of an Inducible P*_tet_* Promoter

The construction of the P*_fliC_*-*gfp*[AAV], P*_xaxAB_*-*gfp*[AAV], P*_xhlBA_*-*gfp*[AAV] and P_D31_-*gfp*[AAV] fusions have been described elsewhere [23]. We used a similar method to obtain plasmids expressing the reporter gene *gfp*[AAV] under the control of the *flhD*, *flgB* or *fliL* promoter region. Briefly, DNA fragments corresponding to the *flhD* (576 bp), *flgB* (400 bp) and *fliL* (223 bp) promoters were amplified by PCR from F1 genomic DNA, with primers containing an *Eco*RI or *Bam*HI restriction site. The PCR products were digested and inserted into the corresponding sites of pPROBE′-*gfp*[AAV] for *flhD* and pPROBE-*gfp*[AAV] for *flgB* and *fliL*, yielding P*_flhD_*-*gfp*[AAV], P*_flgB_*-*gfp*[AAV] and P*_fliL_*-*gfp*[AAV].

For P*_tet_*-MCS construction, we amplified the P_Ltet o-1_-MCS-*tetR* DNA fragment from pSS012 by PCR with the Ptet-XhoI-f and 3′PROTetSeq primers and inserted it in place of *gfp*[AAV] in pPROBE-*gfp*[AAV] digested with *Sal*I and *Eco*RV. We amplified the *fliZ* gene by PCR from F1 genomic DNA, with the LfliZ-Eco and RfliZ-Bam primers, and inserted it into P*_tet_*-MCS digested with *Eco*RI and *Bam*HI, to generate P*_tet_*-*fliZ*. The same strategy was used with *flhDC* operon using LflhDEco2 and RflhCBam primers to generate P*_tet_*-*flhDC.* Finally, the P*_tet_*-*fliZ*-*tetR* and P*_tet_*-*flhDC-tetR* DNA fragments were amplified by PCR from P*_tet_*-*fliZ* or P*_tet_*-*flhDC* respectively with the Tet-fliZ-f and Tet-fliZ-r primers and inserted into P*_fliC_*-*gfp*[AAV] digested with *Sal*I and *Sbf*I, to yield the P*_fliC_*-*gfp*[AAV] - P*_tet_*-*fliZ* construct or P*_fliC_*-*gfp*[AAV] - P*_tet_*-*flhDC* construct.

### Quantification of *fliC* Expression in Bacterial Populations

Wild type, *fliA* and *fliZ* strains carrying either P*_fliC_*-*gfp*[AAV] or P*_fliC_*-*gfp*[AAV] – P*_tet_*-*fliZ* constructs were cultured in black-sided, clear-bottomed 96-well plates (Greiner). For each well, 20 µl of a 1/50 dilution of an overnight culture was added to 180 µl of LB supplemented with kanamycin, and 200 ng.ml^−1^ of aTc when required. Then, the plates were incubated, with shaking on an orbital shaker, at 28°C, in an Infinite M200 microplate reader (Tecan). Absorbance at 600 nm and GFP fluorescence intensity, with excitation at 485±4.5 nm and emission at 520±10 nm, were measured after 15 hours of growth. Specific fluorescence was obtained by dividing fluorescence units by the absorbance value. As a control, we checked that addition of aTc did not affect the expression of P*_fliC_*-*gfp*[AAV] fusion in the wild type strain (data not shown).

### Quantification of Gene Expression in Individual Bacterial Cells by Flow Cytometry

Bacterial strains were grown in LB supplemented with kanamycin at 28°C. If necessary, aTc was added at the indicated concentrations after 3 h of growth (OD_540_∼0.1). At an OD_540_ of ∼0.5 (or OD_540_ = 1.8 for P*_xaxAB_*-*gfp*[AAV] and P*_xhlBA_*-*gfp*[AAV] plasmids), samples were taken, washed once with PBS, diluted and immediately analyzed by flow cytometry (FACS Canto II, BD Biosciences). For kinetic analyses, samples were taken at the indicated time points, washed once with PBS and bacteria were fixed by incubation in 2% formaldehyde in PBS for 15 minutes at room temperature. The cells were then washed once with PBS and bacterial pellets were stored at 4°C until flow cytometry analysis. Forward scatter (FSC), side scatter (SSC) and GFP parameters were set to log and bi-exponential display was used for the GFP parameter. We captured a total of 30,000 bacteria for each sample, unless otherwise indicated, and raw data were analyzed with FlowJo version 8.8.6 software (TreeStar).

### Enzyme-Linked Immunosorbent Assay (ELISA)

FliC was detected by ELISA in bacterial lysates of wild type F1, *fliA* and *fliZ* strains carrying either P*_tet_*-*fliZ* or the vector control P*_tet_*-MCS. These strains were cultured in LB medium, and 200 ng.ml^−1^ of anhydrotetracycline (aTc, Clontech) was added, when required, during the early exponential growth phase (OD_540_ = 0.2). Two hours after induction, samples were taken, centrifuged and the bacterial pellets were resuspended in ultrapure water and lysed by three freeze–thaw cycles and sonication. For ELISA, microtiter plates (Maxisorp Nunc-Immuno Plate) were coated with an amount of culture supernatant (50 µl/well) equivalent to 0.1 OD_540_ units of bacterial lysate. The plates were incubated overnight at room temperature, washed three times with 0.05% Tween 20 in PBS (PBS-T) and blocked by incubation with 0.25% BSA in PBS-T for 2 h at room temperature. Anti-FliC antibodies [60] diluted in 1% BSA in PBS-T (1/500) were added to each well and the plates were incubated for 1 h at room temperature. The plates were washed four times with PBS-T and incubated for 1 h with peroxidase-linked donkey anti-rabbit IgG (1/5000 dilution; GE Healthcare). The plates were washed four times with PBS-T, and 100 µl of 1-Step Ultra TMB-ELISA (Pierce) solution was added to each well. Color development was stopped after 20 minutes, by adding 100 µl of 2 M H_2_SO_4_, and absorbance at 450 nm was measured with a microplate reader (Tecan Infinite 200).

### Fluorescence-Activated Cell Sorting (FACS) Analysis and Subpopulation Sorting

For the sorting of GFP-negative and GFP-positive subpopulations, an overnight culture of the F1 strain containing P*_flgB_*-*gfp*[AAV] was washed once with PBS, diluted 1∶250 in fresh LB broth supplemented with kanamycin and incubated at 28°C for 6 h (OD_540_ of 0.3 to 0.8). The cells were then washed once with PBS before FACS analysis.

Flow-cytometric sorting was performed on a FACSAria II cell sorter system (Becton Dickinson). We sorted 10^7^ GFP-negative and GFP-positive cells at 4°C, for a maximum of 2 hours. The sorting efficiency for three independent biological samples was determined by analyzing the GFP fluorescence patterns of the subpopulations obtained. Immediately after cell sorting, RNA Protect Bacteria Reagent (Qiagen) was added to the cells and RNA was extracted with the RNeasy micro kit (Qiagen) as described above and eluted in a final volume of 15 µl.

## Supporting Information

Figure S1 Comparison between RT-qPCR and RNASeq data. The correlation factor (R^2^) between RT-qPCR and RNASeq were calculated from the log_2_fold change ratio (*fliZ* mutant/WT) obtained by RNASeq against the log_2_fold change ratio obtained by RT-qPCR for the 15 genes tested in Table 1.
(PDF)

Figure S2 Survival plot of *Spodoptera littoralis* insect larvae after the injection of wild type (dotted line) and *fliZ, fliAZ and flhD* mutants (spaced dots, dashed and solid lines respectively) of *X. nematophila*.
(PDF)

Figure S3 Bimodal expression of flagellin and hemolysin genes in *Xenorhabdus*. The strains indicated were grown in LB medium to mid-exponential growth phase (strains with P*_fliC_*-*gfp*[AAV] or P_D31_-*gfp*[AAV] constructs) or mid-stationary growth phase (strains with P*_xaxAB_*-*gfp*[AAV] or P*_xhlBA_*-*gfp*[AAV] constructs) and culture samples were observed by fluorescence microscopy. Pictures were taken either in phase-contrast (left panels) or GFP fluorescence illumination (right panels). Bars represent 3 µm.
(TIF)

Figure S4 Effect of FlhDC production on the level of *fliC* gene expression in individual cells of *Xenorhabdus*. The *flhD* strain carrying a P*_fliC_*-*gfp*[AAV] - P*_tet_*-*flhDC* construct was grown in LB and the final concentration of aTc indicated was added when the OD_540_ reached 0.1. Three hours after aTc addition, bacteria were collected and GFP fluorescence signal was recorded in individual cells by flow cytometry. Data are shown on graphs consisting of five two-dimensional dot plots with the GFP signal on the x-axis and the forward scatter parameter (FSC) on the y-axis. Gates corresponding to GFP-negative and GFP-positive populations are indicated and the percentages of GFP-positive cells are indicated on the right, for each sample.
(PDF)

Table S1 Differentially expressed genes between *X. nematophila* strain F1 and its isogenic mutant ΩfliZ (ajusted p-value≤0.05; |log_2_ fold change|≥1). Co-regulated genes that cluster in regions of at least three adjacent genes are colored in green (log_2_ fold change ≤-1; gene down-regulated in mutant ΩfliZ) or red (log_2_ fold change ≥1; gene up-regulated in mutant ΩfliZ).
(PDF)

Table S2 Bacterial strains and plasmids used in this study.
(PDF)

Table S3 Oligonucleotides used in this study.
(PDF)
