# Supplementary figures and images for: FliZ Is a Global Regulatory Protein Affecting the Expression of Flagellar and Virulence Genes in Individual Xenorhabdus nematophila Bacterial Cells
Source: PLoS Genet. 2013 Oct 31;9(10):e1003915. doi: 10.1371/journal.pgen.1003915 (PMC3814329; doi:10.1371/journal.pgen.1003915)

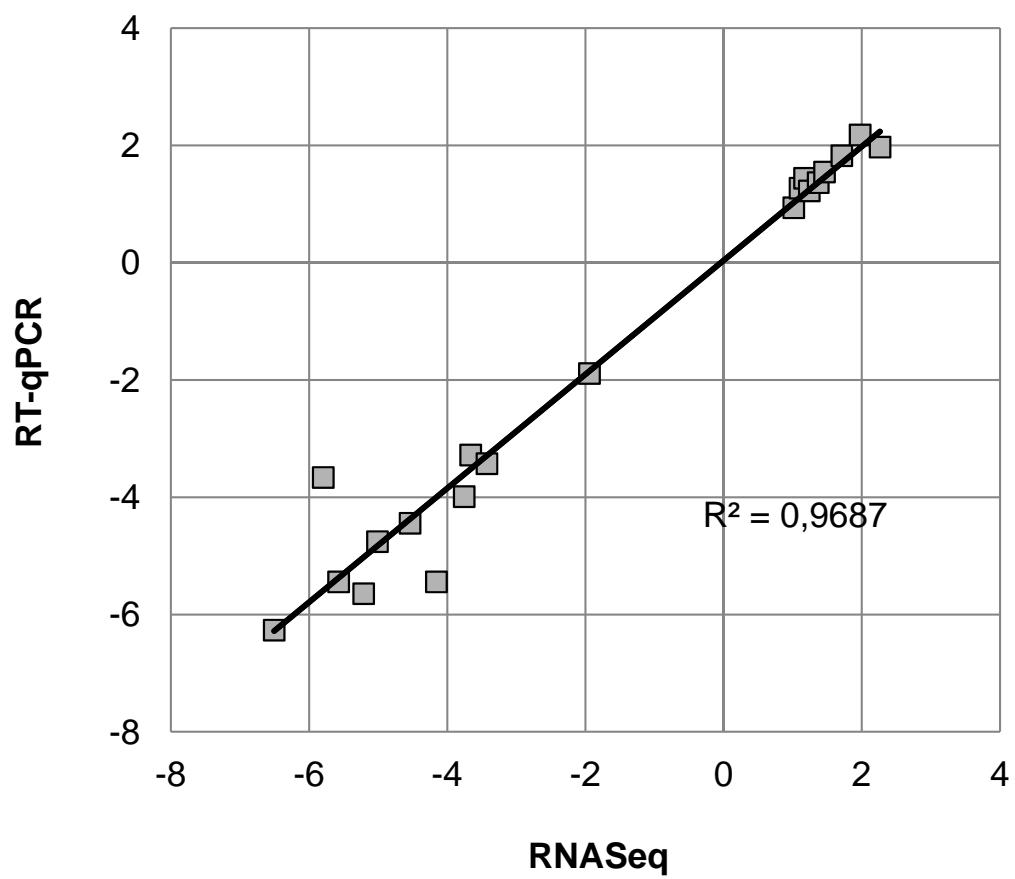

Supplement: Figure S1 — Comparison between RT-qPCR and RNASeq data. The correlation factor (R2) between RT-qPCR and RNASeq were calculated from the log2fold change ratio (fliZ mutant/WT) obtained by RNASeq against the log2fold change ratio obtained by RT-qPCR for the 15 genes tested in Table 1. (PDF) [file pgen.1003915.s001.pdf]

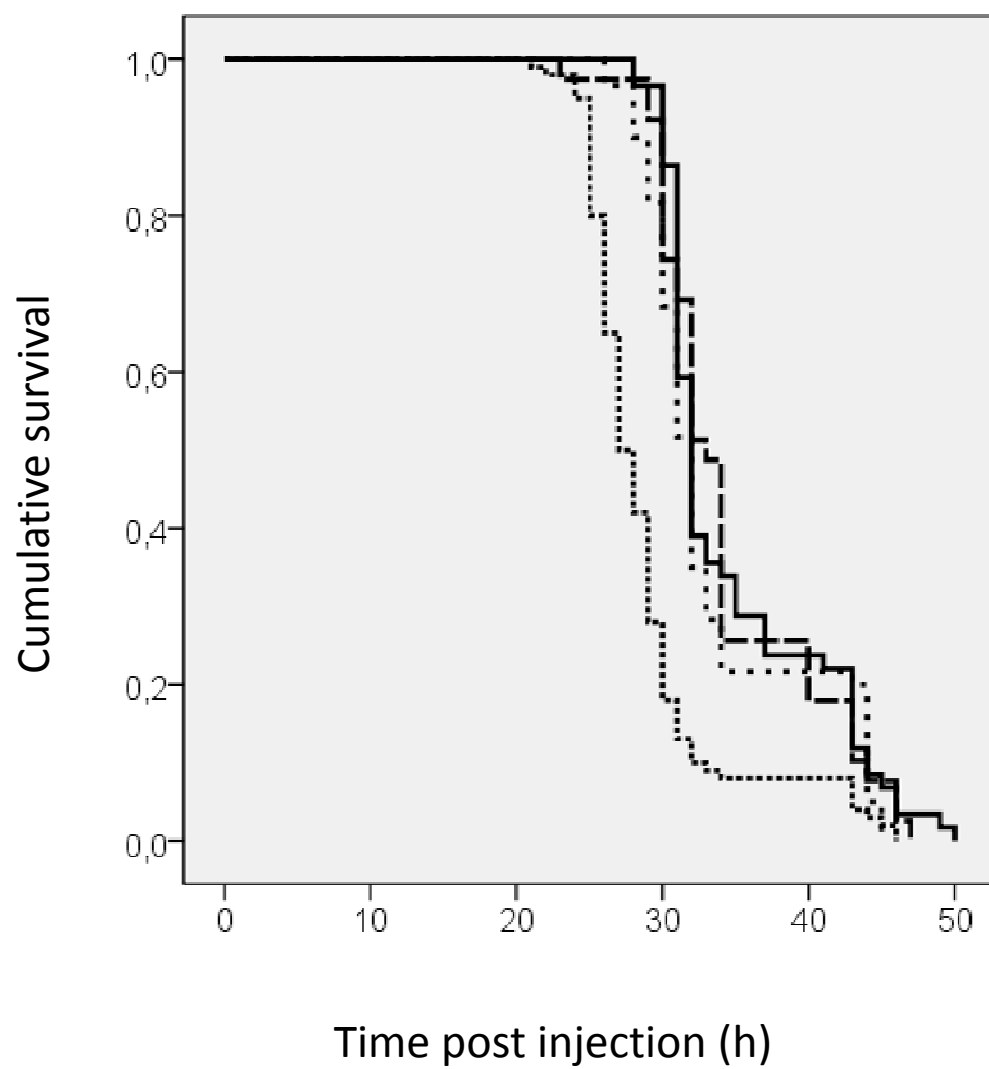

Supplement: Figure S2 — Survival plot of Spodoptera littoralis insect larvae after the injection of wild type (dotted line) and fliZ, fliAZ and flhD mutants (spaced dots, dashed and solid lines respectively) of X. nematophila. (PDF) [file pgen.1003915.s002.pdf]

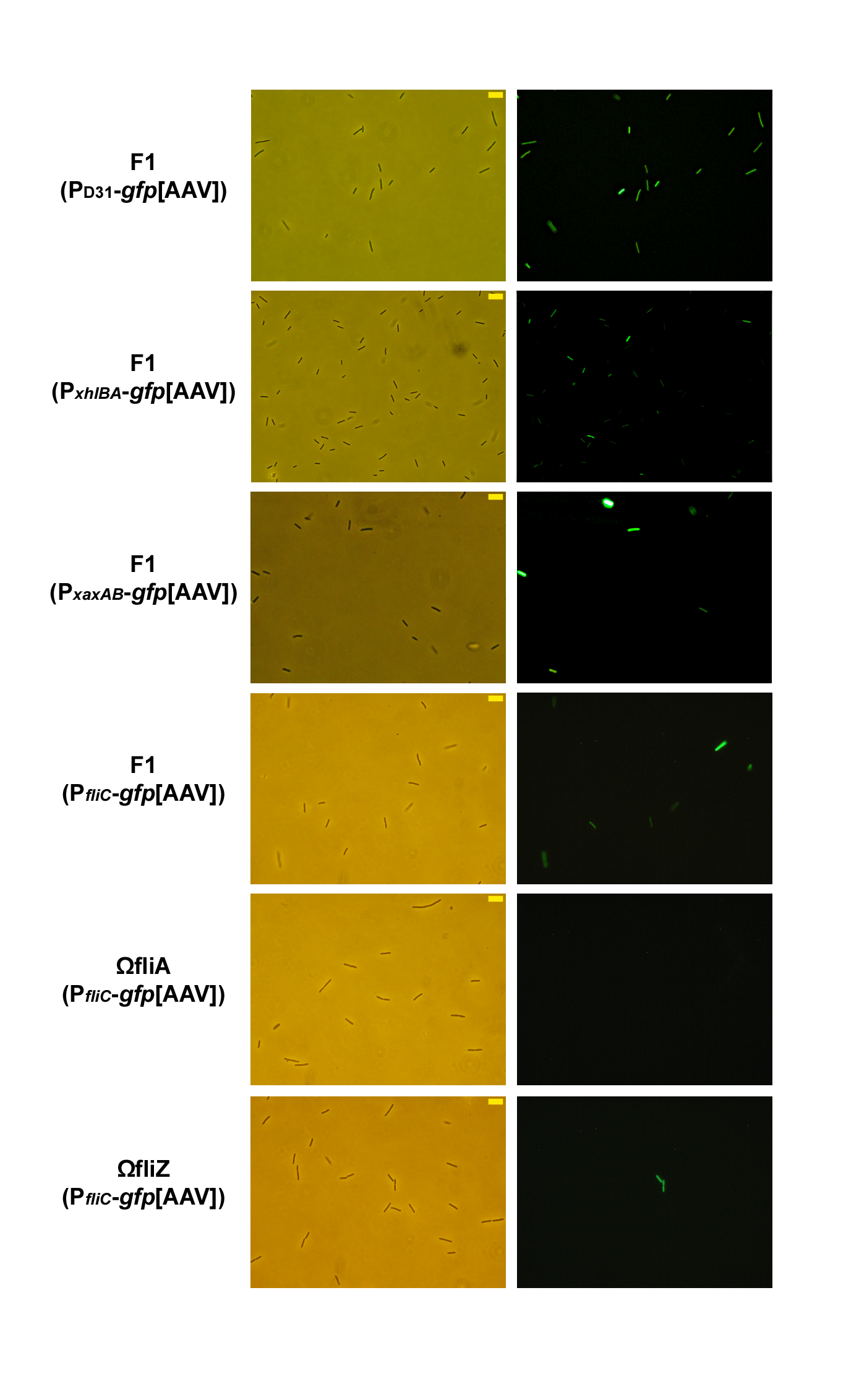

Supplement: Figure S3 — Bimodal expression of flagellin and hemolysin genes in Xenorhabdus. The strains indicated were grown in LB medium to mid-exponential growth phase (strains with PfliC-gfp[AAV] or PD31-gfp[AAV] constructs) or mid-stationary growth phase (strains with PxaxAB-gfp[AAV] or PxhlBA-gfp[AAV] constructs) and culture samples were observed by fluorescence microscopy. Pictures were taken either in phase-contrast (left panels) or GFP fluorescence illumination (right panels). Bars represent 3 µm. (TIF) [file pgen.1003915.s003.tif]

$\Omega$ flhD  
( $P_{fliC}$ -gfp[AAV] -  $P_{tet}$ -flhDC)

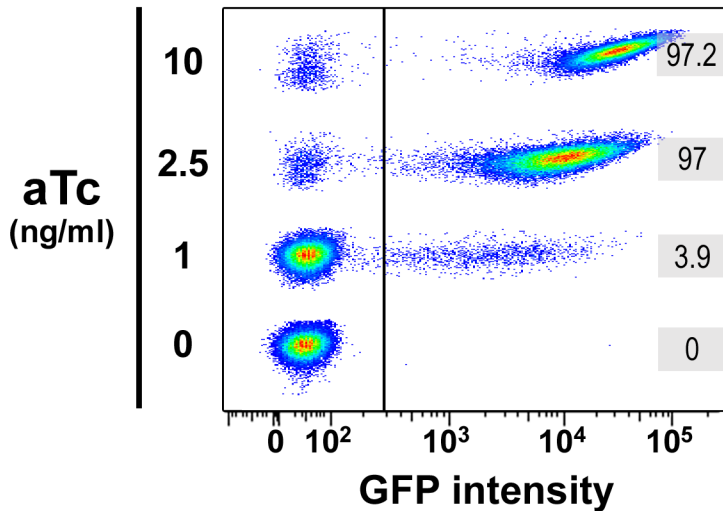

Supplement: Figure S4 — Effect of FlhDC production on the level of fliC gene expression in individual cells of Xenorhabdus. The flhD strain carrying a PfliC-gfp[AAV] - Ptet-flhDC construct was grown in LB and the final concentration of aTc indicated was added when the OD540 reached 0.1. Three hours after aTc addition, bacteria were collected and GFP fluorescence signal was recorded in individual cells by flow cytometry. Data are shown on graphs consisting of five two-dimensional dot plots with the GFP signal on the x-axis and the forward scatter parameter (FSC) on the y-axis. Gates corresponding to GFP-negative and GFP-positive populations are indicated and the percentages of GFP-positive cells are indicated on the right, for each sample. (PDF) [file pgen.1003915.s004.pdf]
