# Supplementary material for: FliZ Is a Global Regulatory Protein Affecting the Expression of Flagellar and Virulence Genes in Individual Xenorhabdus nematophila Bacterial Cells
Source: PLoS Genet. 2013 Oct 31;9(10):e1003915. doi: 10.1371/journal.pgen.1003915 (PMC3814329; doi:10.1371/journal.pgen.1003915)
Supplement: Table S1 — Differentially expressed genes between X. nematophila strain F1 and its isogenic mutant ΩfliZ (ajusted p-value≤0.05; |log2 fold change|≥1). Co-regulated genes that cluster in regions of at least three adjacent genes are colored in green (log2 fold change ≤-1; gene down-regulated in mutant ΩfliZ) or red (log2 fold change ≥1; gene up-regulated in mutant ΩfliZ). (PDF) [file pgen.1003915.s005.pdf]

Table S1. Differentially expressed genes between *X. nematophila* strain F1 and its isogenic mutant Ω*fliZ* (ajusted p-value ≤ 0.05; |log2 fold change|≥ 1). Co-regulated genes that cluster in regions of at least three adjacent genes are colored in green (log2 fold change ≤ -1; gene down-regulated in mutant ΩfliZ) or red (log2 fold change ≥ 1; gene up-regulated in mutant ΩfliZ).

									mutant ΩfliZ/F1 wild-type strain		
**locus number**	**Label**	**Type**	**Gene name**	**Product**	**Begin**	**End**	**Length**	**Frame**	**normalized average read count**	**log2 fold change**	**adjusted p-value**
	XNC3_110002	CDS	-	putative Amino-acid binding protein	154181	155152	972	2	240	1,17	9,52E-05
1	XNC3_110007	CDS	-	Major facilitator superfamily MFS_1	159344	160576	1233	2	1437	1,04	2,57E-05
	XNC3_110008	CDS	-	conserved protein of unknown function	160577	160741	165	2	75	1,08	8,52E-03
	XNC3_110009	CDS	-	protein of unknown function	161065	161181	117	1	26	3,62	7,62E-09
	XNC3_110010	CDS	*nilQ*	NilQ	161739	161963	225	3	8119	1,12	2,52E-09
	XNC3_110011	CDS	*nilR*	NilR transcription factor	162034	162339	306	1	1,23E+04	1,15	6,60E-10
	XNC3_110012	CDS	-	conserved protein of unknown function	162437	162592	156	-3	663	1,18	6,96E-05
	XNC3_120008	CDS	-	conserved exported protein of unknown function	186200	186451	252	-3	984	1,03	2,89E-04
2	XNC3_160008	CDS	-	putative membrane protein	280542	280808	267	-2	62	-1,86	1,18E-05
	XNC3_160009	CDS	-	putative transcriptional repressor for ribose metabolism (GalR/LacI family)	281258	282265	1008	-3	1717	-1,1	2,71E-07
	XNC3_160010	CDS	*ydfJ*	putative inner membrane metabolite transport protein YdfJ	282344	283741	1398	-3	4142	-1,25	9,80E-14
	XNC3_190022	CDS	-	Pyoverdine biosynthesis protein	418279	419286	1008	-1	2,16E+04	-1,01	5,41E-11
	XNC3_190026	CDS	*eco*	Ecotin	422160	422636	477	-2	1355	-1,13	4,85E-07
	XNC3_200020	CDS	-	conserved protein of unknown function	448217	448921	705	-3	7872	-1,42	3,10E-21
	XNC3_250005	CDS	-	conserved exported protein of unknown function	503771	504052	282	2	80	-1,27	3,92E-03
	XNC3_260009	CDS	*pdhR*	Pyruvate dehydrogenase complex repressor	542741	543505	765	-3	4200	-1,05	6,37E-10
	XNC3_290001	CDS	*rpoS*	sigma S (sigma 38) factor of RNA polymerase, major sigma factor during stationary phase	619888	620883	996	-1	1,33E+04	1,15	5,11E-10
	XNC3_350013	CDS	-	conserved protein of unknown function	680520	680702	183	-2	407	-2,53	3,22E-23
3	XNC3_390020	CDS	-	conserved protein of unknown function	744235	744354	120	1	132	-2,43	8,32E-13
	XNC3_390021	CDS	*kamA*	L-lysine 2,3-aminomutase	744410	745588	1179	2	3450	-2,67	4,69E-60
	XNC3_390022	CDS	-	Aminotransferase, class I and II	745622	746791	1170	2	3555	-2,29	9,24E-45
	XNC3_390023	CDS	-	putative Clavaminate synthase	746817	747779	963	3	3331	-2,1	1,02E-36
	XNC3_390024	CDS	-	Long-chain-fatty-acid--CoA ligase (polyketide synthase)	747823	752757	4935	1	9034	-1,61	2,84E-26
	XNC3_390025	CDS	-	Phenylalanine racemase (ATP-hydrolyzing)(polyketide synthase)	752762	758443	5682	2	9744	-1,01	3,84E-10
	XNC3_390032	CDS	*znuC*	Zinc import ATP-binding protein ZnuC	766962	767720	759	-2	1832	-1,34	6,50E-12
4	XNC3_460003	CDS	-	protein of unknown function	838961	839383	423	2	419	-2,11	1,05E-14
	XNC3_470001	fCDS	-	fragment of peptide synthetase (part 1)	839486	841234	1749	2	1707	-1,73	7,19E-18
	XNC3_480001	fCDS	-	fragment of peptide synthetase (part 2)	841411	843300	1890	1	1959	-1,58	8,82E-16
	XNC3_480002	CDS	-	Peptide synthetase	843297	847961	4665	3	4756	-1,49	4,38E-20
	XNC3_480003	fCDS	-	fragment of peptide synthetase (part 1)	847958	850804	2847	2	2043	-1,23	1,03E-09
	XNC3_490001	fCDS	-	fragment of peptide synthetase (part 2)	850917	853493	2577	3	1680	-1,15	1,26E-07
	XNC3_520003	CDS	-	conserved protein of unknown function	904330	904923	594	-1	3484	-1,36	3,73E-15
5	XNC3_540002	CDS	-	putative Peptide synthetase	922392	927353	4962	-2	8921	-1,19	3,60E-14
	XNC3_550001	CDS	-	peptide synthetase (fragment)	927477	928427	951	-2	2397	-1,32	3,13E-12
	XNC3_550002	CDS	-	putative Peptide synthetase	928424	932467	4044	-3	1,07E+04	-1,33	3,45E-18
	XNC3_670002	CDS	-	conserved protein of unknown function	1066743	1067480	738	-2	4132	-1,8	4,08E-29
	XNC3_670003	CDS	-	conserved protein of unknown function	1067484	1067609	126	3	231	-1,77	4,34E-09
	XNC3_700022	CDS	-	conserved protein of unknown function	1137241	1138104	864	-1	3,56E+04	-1,28	1,95E-17
	XNC3_740002	CDS	*xptA1*	A component of insecticidal toxin complex (Tc)	1208948	1216519	7572	2	2,95E+04	-3,41	2,14E-129
	XNC3_740005	CDS	*xptA2*	A component of insecticidal toxin complex (Tc)	1224257	1231873	7617	-3	1,95E+04	-1,04	3,06E-11
	XNC3_800038	CDS	-	conserved exported protein of unknown function	1287616	1288239	624	1	1539	-1	1,78E-05
	XNC3_860010	CDS	-	Similar to photopexin A/B of Photorhabdus luminescens	1368049	1369053	1005	-1	669	-1,11	6,03E-05
	XNC3_860013	CDS	-	conserved protein of unknown function	1370523	1370678	156	-2	449	-1,49	4,13E-08
	XNC3_860014	CDS	-	O-acetylhomoserine (thiol)-lyase	1370747	1372021	1275	2	1,29E+04	-1,52	2,39E-24
	XNC3_860025	CDS	-	conserved protein of unknown function	1381484	1382290	807	-3	2610	-1	2,98E-07
	XNC3_860027	CDS	-	putative 3-oxoacyl-[acyl-carrier-protein] synthase III (Beta-ketoacyl-ACP synthase III) (KAS III)	1383299	1384405	1107	-3	4225	-1,05	1,77E-09
	XNC3_870012	CDS	*xaxA*	Hemolysin component XaxA	1396057	1397271	1215	1	3,71E+04	-6,44	0
	XNC3_870013	CDS	*xaxB*	Hemolysin component XaxB	1397312	1398364	1053	2	4,08E+04	-6,36	0
	XNC3_870020	CDS	-	putative transcriptional regulator, TetR family	1401509	1402147	639	-3	2177	2,21	2,69E-20
	XNC3_920051	CDS	-	PalA	1493803	1506597	12795	-1	1,59E+04	-1,15	1,74E-13

	XNC3_920052	CDS	*hecB*	Hemolysin activator protein	1506665	1508401	1737	-3	2563	-1,67	1,92E-20
	XNC3_930001	CDS	-	protein of unknown function	1521222	1521578	357	-2	269	-1,21	1,24E-04
	XNC3_960004	CDS	-	protein of unknown function	1531386	1531712	327	3	2,16E+04	-2,4	6,20E-63
**6**	XNC3_1090003	CDS	-	conserved protein of unknown function	1631236	1632738	1503	1	5,27E+04	1,2	4,34E-11
	XNC3_1090004	CDS	-	conserved protein of unknown function	1632828	1633775	948	3	7739	1,52	8,72E-16
	XNC3_1090005	CDS	-	conserved protein of unknown function	1633796	1634194	399	2	3301	1,48	1,34E-12
**7**	XNC3_1130016	CDS	-	putative Rhs family protein	1683555	1687952	4398	-2	3,57E+04	-1,12	2,43E-13
	XNC3_1130017	CDS	-	conserved hypothetical protein	1687965	1688384	420	-2	4696	-1,23	4,22E-14
	XNC3_1130018	CDS	-	putative VrgG protein	1688483	1690570	2088	-3	1,53E+04	-1,28	2,25E-17
	XNC3_1220001	CDS	-	putative DNA methylase	1761085	1762182	1098	-1	556	-1,69	2,50E-13
	XNC3_1220005	CDS	-	Transcriptional regulator, XRE family	1765819	1766034	216	-1	70	-1,03	3,28E-03
	XNC3_1220007	CDS	-	protein of unknown function	1767001	1767120	120	-1	468	-1,18	1,09E-06
**8**	XNC3_1220009	CDS	-	protein of unknown function	1768017	1768139	123	3	109	-1,24	7,40E-05
	XNC3_1220010	CDS	-	protein of unknown function	1768117	1768350	234	-1	343	-1,2	1,93E-06
	XNC3_1220011	CDS	-	conserved protein of unknown function	1768200	1769000	801	3	1201	-1,22	2,10E-09
	XNC3_1220012	CDS	-	putative protein p51	1769003	1770292	1290	2	1883	-1,24	1,32E-11
	XNC3_1220013	CDS	-	putative protein p50	1770323	1770880	558	2	917	-1,25	5,75E-09
	XNC3_1220014	CDS	-	conserved protein of unknown function	1770931	1771563	633	1	358	-1,06	3,73E-05
	XNC3_1220015	CDS	-	conserved protein of unknown function	1771556	1773616	2061	2	1309	-1,02	5,46E-07
	XNC3_1220017	CDS	-	putative nuclease p44	1774093	1774362	270	1	118	-1,42	2,04E-06
	XNC3_1220018	CDS	-	Methyltransferase (fragment)	1774359	1774631	273	3	204	-1,09	9,61E-05
	XNC3_1220019	CDS	-	conserved protein of unknown function	1774644	1775435	792	3	343	-1,17	4,01E-06
	XNC3_1220024	CDS	*int*	Integrase	1779264	1780391	1128	3	311	-1,24	9,03E-06
	XNC3_1230001	fCDS	-	fragment of Non-ribosomal peptide synthase involved in xenematides synthesis (part 1)	1791458	1794373	2916	2	2953	-1,48	9,27E-17
	XNC3_1240001	fCDS	-	fragment of Non-ribosomal peptide synthase involved in xenematides synthesis (part 2)	1794563	1803073	8511	2	8379	-1,11	5,69E-12
	XNC3_1240009	CDS	-	putative UmoD	1811581	1812060	480	-1	857	-1,22	4,65E-07
**9**	XNC3_1260002	CDS	-	putative ferric enterobactin transport protein (ABC superfamily, atp_bind)	1848517	1849305	789	-1	872	-1,38	1,90E-09
	XNC3_1260003	CDS	-	putative FecCD-family membrane transport protein (Inner membrane permease of iron/siderophore ABC transporter)	1849302	1850387	1086	-2	3534	-1,43	2,95E-18
	XNC3_1260004	CDS	-	conserved exported protein of unknown function	1850407	1851540	1134	-1	4465	-1,41	6,16E-19
	XNC3_1270006	CDS	-	conserved exported protein of unknown function	1859616	1859927	312	-2	310	-2,01	2,27E-13
	XNC3_1270007	CDS	-	conserved hypothetical protein; putative exported protein	1860212	1860529	318	-3	192	-5,68	3,60E-54
**10**	XNC3_1290003	CDS	*paxC*	Peptide synthetase PaxC	1864455	1875233	10779	-2	5,98E+04	-1,1	8,32E-13
	XNC3_1290004	CDS	*paxB*	Peptide synthetase PaxB	1875238	1885218	9981	-1	5,71E+04	-1,23	3,13E-16
	XNC3_1290005	CDS	*paxA*	Peptide synthetase PaxA	1885255	1888494	3240	-1	2,52E+04	-1,19	6,12E-15
	XNC3_1300001	CDS	*paxT*	putative ATP-binding protein PaxT	1889269	1890894	1626	-1	1,90E+04	-1,36	2,00E-19
	XNC3_1360001	CDS	-	putative lipoprotein	1944834	1945109	276	3	2031	-1,68	2,01E-19
	XNC3_1360002	CDS	-	protein of unknown function	1945361	1945498	138	2	214	-1,68	1,50E-08
	XNC3_1420006	CDS	-	conserved protein of unknown function	2003470	2003826	357	-1	844	-1,01	7,68E-05
	XNC3_1420008	CDS	-	VgrG (fragment)	2005052	2005525	474	-3	1471	-1,02	4,24E-06
	XNC3_1420009	CDS	-	protein of unknown function	2005493	2005624	132	2	271	-1,03	1,35E-03
	XNC3_1460053	CDS	-	conserved membrane protein of unknown function	2087976	2088791	816	-2	5865	-1,25	3,21E-15
	XNC3_1460059	CDS	-	putative Nonribosomal peptide synthase (NRPS)	2096696	2099755	3060	-3	1,14E+04	-1,61	5,07E-27
	XNC3_1530004	CDS	*yfcH*	Epimerase family protein YfcH	2171131	2172057	927	1	4841	1,3	6,75E-11
	XNC3_1590002	CDS	-	putative ferredoxin	2232306	2232746	441	3	333	1,17	1,34E-04
**11**	XNC3_1640031	CDS	-	Phage-related protein	2281793	2282146	354	2	73	1,65	1,47E-05
	XNC3_1640032	CDS	-	protein of unknown function	2282225	2282476	252	2	40	2,04	7,25E-06
	XNC3_1640033	CDS	-	Uncharacterized 8.2 kDa protein in gpA 5\'region	2282469	2282690	222	3	42	2,17	1,59E-06
**12**	XNC3_1640041	CDS	-	conserved protein of unknown function	2289805	2290635	831	1	742	3,04	1,03E-22
	XNC3_1640042	CDS	-	putative phage gene	2290653	2291720	1068	3	976	2,74	2,06E-21
	XNC3_1640043	CDS	-	conserved protein of unknown function	2291720	2292430	711	2	583	2,09	7,85E-13
	XNC3_1640044	CDS	-	putative phage gene	2292427	2292981	555	1	327	2,15	7,87E-12
	XNC3_1640045	CDS	-	putative phage gene	2292978	2293478	501	3	286	1,96	6,52E-10
	XNC3_1640046	CDS	-	putative phage gene	2293475	2294149	675	2	367	1,9	5,07E-10
	XNC3_1640047	CDS	-	putative tail sheath protein	2294172	2295293	1122	3	811	1,92	2,99E-12
	XNC3_1640048	CDS	-	putative tail tube protein	2295290	2295745	456	2	368	1,68	4,20E-08
	XNC3_1640049	CDS	*Y*	Holin	2295758	2296051	294	2	338	1,38	6,88E-06

	XNC3_1640050	CDS	-	conserved protein of unknown function	2296048	2296386	339	1	342	1,25	6,19E-05
	XNC3_1640051	CDS	-	conserved exported protein of unknown function	2296383	2296775	393	3	228	1,22	1,40E-04
	XNC3_1640052	CDS	-	conserved exported protein of unknown function	2296591	2296884	294	1	158	1,21	4,67E-04
	XNC3_1640053	CDS	-	putative phage gene	2296877	2297146	270	2	192	1,54	1,38E-06
	XNC3_1640103	CDS	-	protein of unknown function	2345452	2345589	138	1	34	2,84	1,26E-07
	XNC3_1650007	CDS	-	putative 3-oxoacyl-[acyl-carrier-protein] synthase	2386079	2387125	1047	-3	8347	-1,42	2,46E-20
**13**	XNC3_1720003	CDS	*cheZ*	Protein phosphatase CheZ	2403359	2404015	657	-3	9218	-3,32	1,07E-118
	XNC3_1720004	CDS	*cheY*	Chemotaxis protein CheY	2404025	2404414	390	-3	5224	-3,55	5,54E-123
	XNC3_1720005	CDS	*cheB*	Chemotaxis response regulator protein-glutamate methylesterase	2404496	2405548	1053	-3	6911	-3,73	2,47E-141
	XNC3_1720006	CDS	*cheR*	Chemotaxis protein methyltransferase	2405541	2406428	888	-2	4863	-3,8	3,00E-135
	XNC3_1720007	CDS	*tas*	Methyl-accepting chemotaxis aspartate transducer	2406444	2408066	1623	-2	1,56E+04	-4,09	3,46E-178
	XNC3_1720008	CDS	*tse*	Methyl-accepting chemotaxis serine transducer	2408123	2409826	1704	-3	2,27E+04	-4,52	1,44E-213
	XNC3_1720009	CDS	-	conserved protein of unknown function	2409852	2409980	129	3	381	-3,23	1,54E-34
	XNC3_1720010	CDS	*cheW*	Chemotaxis protein CheW	2410016	2410513	498	-3	4692	-3,1	4,78E-93
	XNC3_1720011	CDS	*cheA*	Chemotaxis protein CheA	2410608	2412755	2148	-2	1,78E+04	-3,52	1,38E-136
	XNC3_1720012	CDS	*motB*	Motility protein B	2412762	2413766	1005	-2	9676	-3,76	3,50E-149
	XNC3_1720013	CDS	*motA*	Motility protein A	2413766	2414647	882	-3	8130	-4,08	9,07E-169
	XNC3_1720014	CDS	*flhC*	Flagellar transcriptional regulator FlhC	2414783	2415367	585	-3	1,01E+04	-1,93	4,46E-41
	XNC3_1720015	CDS	*flhD*	Flagellar transcriptional regulator FlhD	2415370	2415720	351	-1	6314	-1,95	7,43E-40
	XNC3_1720016	CDS	-	protein of unknown function	2415977	2416096	120	2	81	-2,49	8,09E-12
	XNC3_1780006	CDS	*slyA*	Transcriptional regulator SlyA	2469710	2470147	438	-3	2347	-3,61	2,16E-93
	XNC3_1790050	CDS	-	conserved hypothetical protein	2527107	2527970	864	3	3561	1,94	1,38E-19
	XNC3_1850005	CDS	-	conserved exported protein of unknown function	2574639	2574779	141	-2	655	-1,22	4,34E-06
	XNC3_1850006	CDS	-	putative E14 prophage; tail fiber protein (Modular protein)	2574721	2575584	864	1	4618	-1,08	1,77E-10
	XNC3_2090005	CDS	-	protein of unknown function	2856934	2857374	441	-1	644	-1,41	9,21E-09
	XNC3_2130012	CDS	-	putative alanine racemase	2876834	2878066	1233	-3	4607	-1,33	2,20E-16
	XNC3_2140041	CDS	-	conserved exported protein of unknown function	2904252	2904761	510	-2	3130	1,04	5,60E-07
	XNC3_2190004	CDS	-	conserved protein of unknown function	2996092	2996778	687	1	4946	-1,25	1,42E-14
	XNC3_2190005	CDS	-	conserved protein of unknown function	2996756	2997187	432	2	884	-1,26	2,98E-07
	XNC3_2240009	CDS	*yhdH*	putative quinone oxidoreductase YhdH	3053076	3054053	978	-2	1,40E+04	-1,35	2,28E-19
	XNC3_2330007	CDS	-	putative permease (MFS superfamily)	3120698	3121969	1272	-3	4266	1,04	1,43E-07
	XNC3_2370001	CDS	-	putative Cytochrome-c oxidase	3178224	3179972	1749	3	5308	-1,51	1,12E-21
**14**	XNC3_2390004	CDS	*fliZ*	flagellar transcriptional regulator	3213076	3213588	513	-1	1,35E+04	-8,47	0
	XNC3_2390005	CDS	*fliA*	RNA polymerase sigma factor for flagellar operon	3213640	3214362	723	-1	2,17E+04	-4,15	1,65E-184
	XNC3_2390006	CDS	*fliC*	Flagellin	3214627	3215568	942	-1	3,06E+05	-5,55	5,23E-296
	XNC3_2390007	CDS	*fliD*	Flagellar hook-associated protein 2	3215844	3217316	1473	3	3,64E+04	-4,26	8,20E-195
	XNC3_2390008	CDS	*fliS*	Flagellar protein FliS	3217329	3217739	411	3	5140	-3,44	1,80E-115
	XNC3_2390009	CDS	*fliT*	Flagellar protein FliT	3217739	3218113	375	2	3397	-2,92	7,24E-75
	XNC3_2390010	CDS	-	conserved protein of unknown function	3218439	3218672	234	3	546	-3,62	6,67E-47
	XNC3_2390011	CDS	-	conserved protein of unknown function	3218699	3219436	738	2	3116	-2,43	4,83E-50
	XNC3_2390012	CDS	*fliE*	Flagellar hook-basal body complex protein FliE	3219858	3220169	312	-2	2908	-3,43	7,68E-94
	XNC3_2390013	CDS	*fliF*	Flagellar M-ring protein	3220453	3222177	1725	1	1,59E+04	-3,69	1,77E-148
	XNC3_2390014	CDS	*fliG*	Flagellar motor switch protein FliG	3222174	3223166	993	3	9525	-3,53	9,25E-133
	XNC3_2390015	CDS	*fliH*	Flagellar assembly protein FliH	3223159	3223863	705	1	5390	-3,52	2,04E-121
	XNC3_2390016	CDS	*fliI*	Flagellum-specific ATP synthase	3223863	3225227	1365	3	5247	-3,36	6,36E-111
	XNC3_2390017	CDS	*fliJ*	Flagellar FliJ protein	3225262	3225708	447	1	1810	-3,14	1,00E-63
	XNC3_2390018	CDS	-	Flagellar hook-length control protein FliK	3225705	3227072	1368	3	5502	-3,19	9,53E-102
	XNC3_2390019	CDS	*fliL*	Flagellar protein FliL	3227291	3227767	477	2	7222	-3,27	2,70E-112
	XNC3_2390020	CDS	*fliM*	Flagellar motor switch protein FliM	3227773	3228780	1008	1	1,24E+04	-3,23	1,31E-115
	XNC3_2390021	CDS	*fliN*	Flagellar motor switch protein FliN	3228773	3229180	408	2	5051	-3,19	2,33E-100
	XNC3_2390022	CDS	*mopB*	Protein MopB	3229183	3229644	462	1	3313	-3,09	1,09E-82
	XNC3_2390023	CDS	*fliP*	Flagellar biosynthetic protein FliP	3229634	3230473	840	2	3865	-2,76	1,48E-70
	XNC3_2390024	CDS	*fliQ*	Flagellar biosynthetic protein FliQ	3230521	3230790	270	1	749	-2,43	2,46E-26
	XNC3_2390025	CDS	-	protein of unknown function	3230780	3231163	384	-3	722	-2,11	9,04E-20
	XNC3_2390026	CDS	*fliR*	Flagellar biosynthetic protein FliR	3230793	3231575	783	3	1149	-2,07	5,86E-23
**15**	XNC3_2390028	CDS	*xcnN*	Fatty acid desaturase involved in xenocoumacin synthesis	3233769	3234851	1083	-2	1797	-1,34	3,75E-11
	XNC3_2390029	CDS	*xcnM*	Saccharopine dehydrogenase involved in xenocoumacin synthesis	3235179	3236264	1086	-2	2702	-1,43	1,86E-15
	XNC3_2390030	CDS	*xcnL*	Polyketide synthase involved in xenocoumacin synthesis	3236412	3240875	4464	-2	1,66E+04	-1,48	3,22E-23

	XNC3_2390031	CDS	*xcnK*	Non-ribosomal peptide synthase involved in xenocoumacin synthesis	3240978	3243551	2574	-2	1,17E+04	-1,43	2,59E-21
	XNC3_2390032	CDS	*xcnJ*	Conserved hypothetical protein involved in xenocoumacin synthesis	3243734	3244057	324	-3	1383	-1,48	4,24E-12
	XNC3_2390033	CDS	*xcnI*	Thioesterase involved in xenocoumacin synthesis	3244066	3244797	732	-1	3648	-1,49	5,53E-19
	XNC3_2390034	CDS	*xcnH*	Polyketide synthase involved in xenocoumacin synthesis	3245003	3250786	5784	-3	3,07E+04	-1,43	1,12E-21
	XNC3_2390035	CDS	*xcnG*	Beta-lactamase class C involved in xenocoumacin synthesis	3250911	3252380	1470	-2	6193	-1,52	7,10E-23
	XNC3_2390036	CDS	*xcnF*	Polyketide synthase involved in xenocoumacin synthesis	3252517	3262782	10266	-1	6,86E+04	-1,48	1,07E-23
	XNC3_2390037	CDS	*xcnE*	Acyl-CoA dehydrogenase involved in xenocoumacin synthesis	3262987	3264138	1152	-1	2,29E+04	-1,53	7,37E-25
	XNC3_2390038	CDS	*xcnD*	Putative acyl carrier protein potentially involved in xenocoumacin synthesis	3264146	3264403	258	-3	4320	-1,46	3,41E-19
	XNC3_2390039	CDS	*xcnC*	Methoxymalonate biosynthesis protein involved in xenocoumacin synthesis	3264437	3265501	1065	-3	1,47E+04	-1,52	2,37E-24
	XNC3_2390040	CDS	*xcnB*	3-hydroxyacyl-CoA dehydrogenase involved in xenocoumacin synthesis	3265501	3266358	858	-1	1,53E+04	-1,51	4,03E-24
	XNC3_2400001	CDS	*xcnA*	Non-ribosomal peptide synthase involved in Xenocoumacin synthesis	3267114	3275147	8034	-2	7,56E+04	-1,49	3,71E-24
	XNC3_2400002	CDS	-	conserved protein of unknown function	3275575	3275904	330	-1	251	-1,15	2,32E-04
**16**	XNC3_2410002	CDS	-	conserved protein of unknown function	3278556	3279440	885	3	2352	1,36	3,84E-10
	XNC3_2410003	CDS	-	putative cysteine desulfurase (TRNA sulfurtransferase), PLP-dependent	3279453	3280607	1155	3	2616	1,33	3,65E-10
	XNC3_2410004	CDS	-	conserved protein of unknown function	3280618	3281442	825	1	3019	1,03	6,31E-07
**17**	XNC3_2410005	CDS	*flgL*	Flagellar hook-associated protein 3	3281770	3282738	969	-1	1,70E+04	-4,29	3,31E-194
	XNC3_2410006	CDS	*flgK*	Flagellar hook-associated protein 1	3282774	3284414	1641	-2	3,12E+04	-4,5	2,38E-213
	XNC3_2410007	CDS	*flgJ*	Peptidoglycan hydrolase FlgJ	3284635	3285612	978	-1	8159	-3,41	5,54E-123
	XNC3_2410008	CDS	*flgI*	Flagellar P-ring protein	3285612	3286739	1128	-2	1,53E+04	-3,68	1,13E-147
	XNC3_2410009	CDS	-	conserved protein of unknown function	3286639	3286785	147	-1	2118	-3,63	3,02E-89
	XNC3_2410010	CDS	*flgH*	Flagellar L-ring protein	3286746	3287537	792	-2	1,29E+04	-3,58	2,88E-139
	XNC3_2410011	CDS	*flgG*	Flagellar basal-body rod protein FlgG	3287604	3288386	783	-2	3,09E+04	-3,75	3,96E-155
	XNC3_2410012	CDS	*flgF*	Flagellar basal-body rod protein FlgF	3288404	3289159	756	-3	3,49E+04	-3,77	1,14E-156
	XNC3_2410013	CDS	*flgE*	flagellar biosynthesis; hook protein	3289196	3290392	1197	-3	5,28E+04	-3,74	2,83E-156
	XNC3_2410014	CDS	*flgD*	Basal-body rod modification protein FlgD	3290408	3291109	702	-3	2,37E+04	-3,74	2,45E-153
	XNC3_2410015	CDS	*flgC*	Flagellar basal-body rod protein FlgC	3291123	3291533	411	-2	1,39E+04	-3,5	5,75E-134
	XNC3_2410016	CDS	*flgB*	Flagellar basal body rod protein FlgB	3291533	3291949	417	-3	1,46E+04	-3,68	2,63E-147
	XNC3_2410017	CDS	*flgA*	Flagella basal body P-ring formation protein FlgA	3292166	3292837	672	2	5451	-2,73	7,70E-76
	XNC3_2410018	CDS	*flgM*	Negative regulator of flagellin synthesis	3292960	3293259	300	1	3308	-2,82	4,03E-69
	XNC3_2410019	CDS	*flgN*	Flagella synthesis protein FlgN	3293286	3293726	441	3	4618	-2,44	1,25E-58
	XNC3_2410025	CDS	*flhA*	Flagellar biosynthesis protein FlhA	3299949	3302036	2088	-2	1,06E+04	-2,69	3,16E-80
	XNC3_2410026	CDS	*flhB*	Flagellar biosynthetic protein FlhB	3302029	3303180	1152	-1	3513	-3,25	1,81E-92
	XNC3_2420011	CDS	-	protein of unknown function	3310265	3310630	366	-3	958	-1,12	9,95E-07
	XNC3_2420012	CDS	-	conserved protein of unknown function	3310630	3311220	591	-1	1581	-1,02	5,74E-07
**18**	XNC3_2420015	CDS	-	Hypothetical bacteriophage protein	3311666	3312196	531	-3	2108	-1,06	1,58E-08
	XNC3_2420016	CDS	-	conserved protein of unknown function	3312193	3312579	387	-1	1902	-1,15	1,37E-09
	XNC3_2420017	CDS	-	conserved protein of unknown function	3312566	3313366	801	-3	3325	-1,06	4,19E-10
	XNC3_2420018	CDS	-	conserved protein of unknown function	3313558	3313911	354	-1	1315	-1,15	4,93E-08
	XNC3_2420019	CDS	-	conserved protein of unknown function	3313990	3314400	411	-1	2455	-1,11	9,03E-10
	XNC3_2420020	CDS	-	conserved protein of unknown function	3314397	3314666	270	-2	1071	-1,17	1,22E-07
	XNC3_2420021	CDS	-	protein of unknown function	3314838	3315017	180	-2	423	-1,08	3,86E-05
	XNC3_2420022	CDS	-	protein of unknown function	3315064	3315333	270	-1	652	-1,11	4,29E-06
	XNC3_2420038	CDS	*hol*	holin	3324936	3325259	324	3	761	-1,11	4,16E-06
	XNC3_2420040	CDS	-	putative Phosphoprotein phosphatase	3325654	3326106	453	1	721	-1,04	2,40E-05
**19**	XNC3_2420045	CDS	-	conserved protein of unknown function	3330387	3331133	747	3	579	-1,07	6,92E-06
	XNC3_2420046	CDS	-	putative phage head protein/prohead protease	3331136	3332344	1209	2	1330	-1,47	9,54E-14
	XNC3_2420047	CDS	-	putative bacteriophage protein	3332349	3332840	492	3	707	-1,67	5,03E-14
	XNC3_2420048	CDS	-	putative bacteriophage protein	3332849	3333787	939	2	1137	-1,76	2,43E-18
	XNC3_2420049	CDS	-	conserved protein of unknown function	3333787	3334134	348	1	444	-1,64	4,88E-12
	XNC3_2420050	CDS	-	conserved protein of unknown function	3334115	3334525	411	2	420	-1,5	4,73E-10
	XNC3_2420051	CDS	-	conserved protein of unknown function	3334522	3335064	543	1	484	-1,6	1,19E-11
	XNC3_2420052	CDS	-	putative head-tail adaptor	3335064	3335444	381	3	417	-1,61	1,81E-11
	XNC3_2420053	CDS	-	putative bacteriophage protein	3335441	3335980	540	2	439	-1,67	2,35E-12
	XNC3_2420054	CDS	-	conserved protein of unknown function	3335986	3337452	1467	1	883	-1,67	5,35E-15
	XNC3_2420055	CDS	-	conserved protein of unknown function	3337457	3337897	441	2	274	-1,56	1,04E-09
	XNC3_2420056	CDS	-	putative bacteriophage protein	3337897	3338319	423	1	315	-1,09	2,79E-05
	XNC3_2420071	CDS	-	conserved protein of unknown function	3348679	3348987	309	-1	106	-1,11	4,99E-03
**20**	XNC3_2450019	CDS	-	Gp10	3373312	3373755	444	-1	275	-1,08	2,79E-04

	XNC3_2450020	CDS	-	putative head-tail adaptor	3373752	3374072	321	-2	179	-1,14	3,33E-04
	XNC3_2450021	CDS	-	conserved protein of unknown function	3374072	3374377	306	-3	221	-1,12	2,91E-04
	XNC3_2450023	CDS	-	Sb5	3375643	3376302	660	-1	498	-1,07	7,96E-05
	XNC3_2450024	CDS	-	putative portal protein	3376274	3377512	1239	-3	804	-1,2	8,58E-07
	XNC3_2450025	CDS	-	conserved exported protein of unknown function	3377512	3377688	177	-1	125	-1,21	5,32E-04
	XNC3_2450026	CDS	-	putative phage terminase, large subunit	3377697	3379433	1737	-2	1293	-1,22	1,75E-08
	XNC3_2450027	CDS	-	conserved protein of unknown function	3379436	3379897	462	-3	527	-1,26	1,15E-06
	XNC3_2450028	CDS	-	protein of unknown function	3379711	3380076	366	-1	397	-1,14	3,33E-05
	XNC3_2450029	CDS	-	conserved protein of unknown function	3380028	3380432	405	-2	521	-1,27	1,02E-06
	XNC3_2450030	CDS	-	conserved protein of unknown function	3380529	3381101	573	-2	587	-1,2	2,39E-06
	XNC3_2490026	CDS	-	conserved protein of unknown function	3410774	3411352	579	2	106	-1,57	4,48E-05
	XNC3_2500005	CDS	*kdpA*	Potassium-transporting ATPase A chain	3452118	3453815	1698	3	1839	1,1	1,79E-06
	XNC3_2530009	CDS	-	conserved protein of unknown function	3529630	3529842	213	-1	28	-1,74	2,57E-03
	XNC3_2530010	CDS	-	conserved membrane protein of unknown function	3529815	3530309	495	-2	224	-1,21	2,03E-04
	XNC3_2530027	CDS	-	conserved protein of unknown function	3545621	3546154	534	2	2,63E+04	-1,35	8,36E-20
	XNC3_2530028	CDS	-	conserved protein of unknown function	3546191	3546364	174	2	2049	-1,18	1,18E-09
	XNC3_2530030	CDS	*feoB*	Ferrous iron transport protein B	3546797	3549112	2316	2	1,19E+04	1,22	1,38E-10
	XNC3_2530031	CDS	*feoC*	Ferrous iron transport protein C	3549128	3549367	240	2	1003	1,15	5,16E-05
**21**	XNC3_2600005	CDS	-	conserved membrane protein of unknown function	3624591	3625742	1152	-2	1,50E+04	-1,04	3,69E-11
	XNC3_2600006	CDS	-	putative Inorganic diphosphatase	3625895	3626548	654	-3	1,52E+04	-1,29	1,69E-17
	XNC3_2600007	CDS	-	putative L-iditol 2-dehydrogenase	3626574	3627635	1062	-2	2,52E+04	-1,41	4,45E-21
	XNC3_2600008	CDS	-	putative 4-aminobutyrate aminotransferase, PLP-dependent	3627635	3628885	1251	-3	2,49E+04	-1,5	4,55E-24
**22**	XNC3_2670009	CDS	*prtA*	Serralysin-like metalloprotease PrtA	3760574	3762010	1437	2	1,26E+05	-4,95	8,80E-254
	XNC3_2670010	CDS	-	Alkaline proteinase inhibitor (fragment)	3762195	3762527	333	3	4387	-3,59	1,84E-118
	XNC3_2670011	CDS	*aprD*	Alkaline protease secretion ATP-binding protein AprD	3762542	3764323	1782	2	1,36E+04	-3,27	3,21E-118
	XNC3_2670012	CDS	*aprE*	Alkaline protease secretion protein AprE	3764375	3765712	1338	2	6950	-2,96	3,42E-92
	XNC3_2670013	CDS	*aprF*	Alkaline protease secretion protein AprF	3765712	3767115	1404	1	4590	-2,3	2,03E-51
	XNC3_2730025	CDS	-	conserved protein of unknown function	3908529	3908696	168	-2	20	-3,1	3,05E-07
	XNC3_2730026	CDS	*ogt*	Methylated-DNA--protein-cysteine methyltransferase	3909036	3909500	465	3	3598	-2,27	2,24E-45
	XNC3_2830004	CDS	-	conserved protein of unknown function	4017364	4017825	462	-1	2139	-1,54	9,26E-16
	XNC3_2830013	CDS	-	conserved protein of unknown function	4029597	4029899	303	-2	46	-2,07	9,72E-06
	XNC3_2830014	CDS	-	conserved protein of unknown function	4029934	4030110	177	-1	12	-2,15	1,13E-02
	XNC3_2860001	CDS	-	conserved protein of unknown function	4079024	4079353	330	-3	234	1,68	3,42E-07
	XNC3_2860002	CDS	-	conserved exported protein of unknown function	4079118	4079441	324	3	279	1,58	1,02E-06
**23**	XNC3_2880003	CDS	*xhlA*	XhlA, Cell surface associated hemolysin (TpsA)	4092926	4097338	4413	-3	6,68E+04	-5,2	6,38E-275
	XNC3_2880004	CDS	*xhlB*	XhlB, XhlA hemolysin secretion/activation protein (TpsB)	4097454	4099127	1674	-2	3503	-4,01	1,24E-131
	XNC3_2880005	CDS	*hcp*	Hemolysin-coregulated protein Hcp, SSTVI secreted cytotoxin system component	4099757	4100275	519	-3	4,40E+04	-1,38	2,60E-20
	XNC3_2880006	CDS	-	putative component of the SST VI cluster	4101206	4101703	498	2	3,35E+04	-1,31	4,52E-18
	XNC3_2880007	CDS	-	putative component of the SST VI cluster	4101723	4103201	1479	3	9,37E+04	-1,28	1,76E-17
	XNC3_2880008	CDS	-	putative component of the SST VI cluster; lysozyme-related protein	4103204	4103644	441	2	1,11E+04	-1,27	1,35E-16
	XNC3_2880009	CDS	-	putative component of the SST VI cluster	4103645	4105477	1833	2	2,59E+04	-1,25	1,78E-16
	XNC3_2880010	CDS	-	putative component of the SST VI cluster	4105441	4106493	1053	1	1,17E+04	-1,21	3,99E-15
	XNC3_2880011	CDS	-	putative component of the SST VI cluster with FHA domain	4106499	4107785	1287	3	1,30E+04	-1,16	7,80E-14
	XNC3_2880012	CDS	-	putative component of the SST VI cluster; lipoprotein	4107769	4108323	555	1	6773	-1,19	6,31E-14
	XNC3_2880013	CDS	-	putative component of the SST VI cluster	4108326	4109678	1353	3	1,57E+04	-1,12	5,27E-13
	XNC3_2880014	CDS	-	putative component of the SST VI cluster	4109681	4110448	768	2	8390	-1,13	1,09E-12
	XNC3_2880015	CDS	-	putative component of the SST VI cluster; ClpA/B-type chaperone	4110458	4113193	2736	2	2,53E+04	-1,12	3,79E-13
	XNC3_2880016	CDS	-	putative component of the SST VI cluster	4113193	4113990	798	1	4928	-1,07	1,75E-10
	XNC3_2880017	CDS	-	putative component of the SST VI cluster	4113987	4114658	672	3	3109	-1,09	1,74E-09
	XNC3_2880018	CDS	-	putative component of the SST VI cluster with ImpA domain	4114664	4116106	1443	2	7868	-1,07	2,46E-11
	XNC3_2880019	CDS	-	putative component of the SST VI cluster	4116103	4119708	3606	1	2,23E+04	-1,12	5,15E-13
	XNC3_2880020	CDS	-	putative component of the SST VI cluster with ImpA domain	4119791	4121317	1527	2	6562	-1,17	2,50E-13
	XNC3_3040001	CDS	-	conserved protein of unknown function	4202056	4202316	261	1	23	-1,38	3,56E-02
	XNC3_3170003	CDS	-	conserved protein of unknown function	4241806	4242165	360	1	7545	-2,93	7,30E-90
	XNC3_3170004	CDS	-	exported protein of unknown function	4242168	4242563	396	3	6946	-2,79	8,42E-81
	XNC3_3220001	CDS	-	hypothetical protein	4251241	4251384	144	1	2004	-1,28	7,41E-11
