# Supplementary material for: FliZ Is a Global Regulatory Protein Affecting the Expression of Flagellar and Virulence Genes in Individual Xenorhabdus nematophila Bacterial Cells
Source: PLoS Genet. 2013 Oct 31;9(10):e1003915. doi: 10.1371/journal.pgen.1003915 (PMC3814329; doi:10.1371/journal.pgen.1003915)
Supplement: Table S2 — Bacterial strains and plasmids used in this study. (PDF) [file pgen.1003915.s006.pdf]

Table S2: Bacterial strains and plasmids used in this study.

Strain or plasmid	Description	Source or reference
***X. nematophila* strain**		
F1	Wild type isolated from *Steinernema carpocapsae* nematode, Plougastel (Brittany)	Laboratory collection
ΩflhD	F1 *flhD*::ΩCm ; *flhD* mutant	[1]
ΩfliA	F1 *fliA*::ΩCm ; *fliAZ* mutant	[2]
ΩfliZ	F1 *fliZ*::ΩCm ; *fliZ* mutant	This work
***E. coli* strain**		
S17.1	*pro* $r^- n^-$ $Tp^R Sm^R$ RP4-2-Tc::Mu::Tn7 *recA thi*	[3]
**Plasmids**		
pJQ200KS	*sacRB mob oriV* (p15A replicon), Gm^R	S. Forst
pHP45-ΩCm	Ap^R Cm^R interposon ΩCm	[4]
pGJ906	pJQ200KS vector containing Ωcam surrounded by upstream and dowstream region of *fliZ*, Gm^R	This work
pSS012	ColE1 vector with $P_{LtetO\text{-}1}$-MCS-*tetR*, Cam^R	[5]
pPROBE-*gfp*[AAV]	Plasmid (pBBR1 replicon) containing *gfp*[AAV] gene downstream from a multiple cloning site, Kan^R	[6]
P_{fliC}-*gfp*[AAV]	pPROBE with *gfp*[AAV] under the control of *fliC* gene promoter, Kan^R	[7]
P_{xaxAB}-*gfp*[AAV]	pPROBE' with *gfp*[AAV] under the control of *xaxAB* gene promoter, Kan^R	[7]
P_{xhlBA}-*gfp*[AAV]	pPROBE' with *gfp*[AAV] under the control of *xhlBA* gene promoter, Kan^R	[7]
P_{flhD}-*gfp*[AAV]	pPROBE' with *gfp*[AAV] under the control of *flhD* gene promoter, Kan^R	This work
P_{flgB}-*gfp*[AAV]	pPROBE with *gfp*[AAV] under the control of *flgB* gene promoter, Kan^R	This work
P_{fliL}-*gfp*[AAV]	pPROBE with *gfp*[AAV] under the control of *fliL* gene promoter, Kan^R	This work
P_{tet}-MCS	pPROBE-*gfp*[AAV] with $P_{LtetO\text{-}1}$-MCS-*tetR* from pSS012 instead of *gfp*[AAV], Kan^R	This work
P_{tet}-*fliZ*	P_{tet}-MCS with *fliZ-tetR* gene under the control of $P_{LtetO\text{-}1}$ promoter, Kan^R	This work
P_{fliC}-*gfp*[AAV] - P_{tet}-*fliZ*	P_{fliC}-*gfp*[AAV] containing $P_{LtetO\text{-}1}$-*fliZ-tetR*, Kan^R	This work

1. Givaudan A, Lanois A (2000) *flhDC*, the flagellar master operon of *Xenorhabdus nematophilus*: requirement for motility, lipolysis, extracellular hemolysis, and full virulence in insects. J Bacteriol 182: 107-115.
2. Lanois A, Jubelin G, Givaudan A (2008) FliZ, a flagellar regulator, is at the crossroads between motility, haemolysin expression and virulence in the insect pathogenic bacterium *Xenorhabdus*. Mol Microbiol 68: 516-533.
3. Simon R, Priefer U, Pühler A (1983) A broad host-range mobilization system for in vivo genetic engineering: transposon mutagenesis in Gram-negative bacteria. Bio/Technology 1: 784-791.
4. Fellay R, Frey J, Krisch H (1987) Interposon mutagenesis of soil and water bacteria: a family of DNA fragments designed for in vitro insertional mutagenesis of gram- negative bacteria. Gene 52: 147-154.
5. Saini S, Brown JD, Aldridge PD, Rao CV (2008) FliZ is a posttranslational activator of FlhD4C2-dependent flagellar gene expression. J Bacteriol 190: 4979-4988.
6. Miller WG, Leveau JH, Lindow SE (2000) Improved gfp and inaZ broad-host-range promoter-probe vectors. Mol Plant Microbe Interact 13: 1243-1250.
7. Jubelin G, Pages S, Lanois A, Boyer MH, Gaudriault S, et al. (2011) Studies of the dynamic expression of the *Xenorhabdus* FliAZ regulon reveal atypical iron-dependent regulation of the flagellin and haemolysin genes during insect infection. Environ Microbiol 13: 1271-1284.
