# Supplementary material for: FliZ Is a Global Regulatory Protein Affecting the Expression of Flagellar and Virulence Genes in Individual Xenorhabdus nematophila Bacterial Cells
Source: PLoS Genet. 2013 Oct 31;9(10):e1003915. doi: 10.1371/journal.pgen.1003915 (PMC3814329; doi:10.1371/journal.pgen.1003915)
Supplement: Table S3 — Oligonucleotides used in this study. (PDF) [file pgen.1003915.s007.pdf]

Table S3. Oligonucleotides used in this study

I. primers used for the construction of *X. nematophila* mutant and plasmids:

**Name**	**Sequence (5' to 3')**	**Location**
fliA-Xba-f	GC**TCTAGA**CGTGATGGACAAAAATAGCCTC	to amplify region upstream *fliZ*
fliA-BamHI-r	CG**GGATCC**GTTACTTCTCTGGATTCAGACGC	to amplify region upstream *fliZ*
putA-BamHI-f	CG**GGATCC**CATCAAGCTGGCATTACCTCC	to amplify region downstream *fliZ*
putA-XhoI-r	CCG**CTCGAG**GTACAACCATTTGGTGGTGAAGG	to amplify region downstream *fliZ*
L-flhDEco-gfp	CG**GAATTC**GGAAGATGAGTGCCTACCTG	to amplify region upstream *flhD*
R-flhDBam-gfp	CG**GGATCC**ATATATCCCGTCCGATTATATG	to amplify region upstream *flhD*
L-flgBBam-gfp	CG**GGATCC**ACACGTTGACCATTGTTGC	to amplify region upstream *flgB*
R-flgBEco-gfp	CG**GAATTC**ATTCCTCCCTCGGTTTTG	to amplify region upstream *flgB*
L-fliLBam-gfp	CG**GGATCC**CCTGATTATCCACCGAGTTATC	to amplify region upstream *fliL*
R-fliLEco-gfp	CG**GAATTC**GGACAGACAAATTCCTGTTTTC	to amplify region upstream *fliL*
Ptet-XhoI-f	GTCTTCACCTCGAGTCCCTATCAG	Used to construct P_{tet}-MCS
3'PROTetSeq	CGCTCGCCGCAGCCGAAC	Used to construct P_{tet}-MCS
L-fliZ-Eco	CG**GAATTC**GGAGGAAAAACATATGTCTGTTACAA CACAAAAG	Used to construct P_{tet}-*fliZ*
R-fliZ-Bam	CG**GGATCC**AATTAATCAATGACTGCTCTGTTGC	Used to construct P_{tet}-*fliZ*
LflhDEco2	CG**GAATTC**TCGGACGGGATATATAGAAATG	Used to construct P_{tet}-*flhDC*
RflhCBam	CG**GGATCC**TGGTGAACAGATGGGATTTG	Used to construct P_{tet}-*flhDC*
tetfliZ-f	ACGC**GTCGAC**TCGAGTCCCTATCAGTGATAGAGA TTG	used to transfer P_{tet}-*fliZ* into P_{fliC}-*gfp*[AAV]
tetfliZ-r	CTGA**CCTGCAGG**GCAGATTTGAGTGAGCTGATAC CG	used to transfer P_{tet}-*fliZ* into P_{fliC}-*gfp*[AAV]

II. primers used for qRT-PCR

**Name**	**Sequence**	**Location**
L-recA	ATTAATACTCTGGGAGAGTTGATCG	Internal region within *recA*
R-recA	AGTTTCTTATTCAACTCAGCAGCAG	Internal region within *recA*
L-16Sq2	ATGCGTAGAGATGTGGAGGAATAC	Internal region within rRNA *16S*
R-16Sq2	CAATTCATTTGAGTTTTAACCTTGC	Internal region within rRNA *16S*
mreBqF	AATTCTGGTGGGCACTGTTC	Internal region within *mreB*
mreBqR	GGATCGGCTTATCCAACAGA	Internal region within *mreB*
L-ampDq	GAAATTGTACAGTACGTTCCTTTCG	Internal region within *ampD*
R-ampDq	CACTTCTGCCAGCTTAATGTATTG	Internal region within *ampD*
5flhDq	CGTTTAGGTATTAGTGAATCGATGG	Internal region within *flhD*
3flhDq	AGATGAGTAGACAGCAAAATACCTG	Internal region within *flhD*
cterfliA-L	AGTGATTGAGGAAGAACATGAAACC	Internal region within *fliA*
cterfliA-R	GGCACCGATCTCTTTAAGATTGAG	Internal region within *fliA*
L-fliZx	GACAAAACCTGGAGTGAATTACAAG	Internal region within *fliZ*
R-fliZx	GACGTCTTAAACGAACCACATACTC	Internal region within *fliZ*
L-flgBxn	GTGATATCGATTTTGCTGCTCAAC	Internal region within *flgB*
R-flgBxn	ATGTCAACGGTATTTCCATCCATAG	Internal region within *flgB*
L-fliL	GTACTAATTGCTGTTATTGGTGCTG	Internal region within *fliL*
R-fliL	AATGTGACCCCAACATATAGAACAC	Internal region within *fliL*
fliCq-F	GGTATCTCCATTGCTCAGACTACC	Internal region within *fliC*
fliCq-R	TGTAGAGATACGGTCAATTTCTTCC	Internal region within *fliC*

Name	Sequence	Location
L-motA	ACTTACGAACAAGAGAGTGAAGTGC	Internal region within *motA*
R-motA	CCAGAGGAGAAACAAAACCATAAG	Internal region within *motA*
L-tse	GCACTGAAAGAACTGGATATATTGC	Internal region within *tse*
R-tse	AATAACACAAACACGGAGATCAGAC	Internal region within *tse*
xaxAq-F	GAGACAGCAGGAAAATACTCAGAAG	Internal region within *xaxA*
xaxAq-R	TGATGGTGGTAGAGAGGTTATTGTC	Internal region within *xaxA*
xhlAquant1	GCTGTTGGCAGATAATTCGCC	Internal region within *xhlA*
xhlAquant2	CGGATGTGAGTCAGGCTGACA	Internal region within *xhlA*
xrtA-L	GGATCTTTAAAGTTTCTGGCTTCAG	Internal region within *xrtA*
xrtA-R	GTATAGACCATTTTCTGACCCTGAG	Internal region within *xrtA*
L-xptA	CGGTGATATATTCCTGTTAAAGCTG	Internal region within *xptA*
R-xptA	GAACATAAAACAGCTTACCCAACAC	Internal region within *xptA*
L-XNC3_870020	AAATCGTTATCTATTGATGCGGTAG	Internal region within XNC3_870020
R-XNC3_870020	GATATTGATGCTCAAACCAATTAGC	Internal region within XNC3_870020
L-XNC3_1790050	ATCGCGACTATAGCTTCCTTTTACT	Internal region within XNC3_1790050
R-XNC3_1790050	GTAGCAAGCATGTTATCAAAATCAA	Internal region within XNC3_1790050
L-XNC3_2410002	TACCAGGGATCATTGAAGAAAATAC	Internal region within XNC3_2410002
R-XNC3_2410002	AGAAGACACGTCTTAGTTCAGATGC	Internal region within XNC3_2410002
L-yfcH	CTGGACCACGTTAAATACTCAACAC	Internal region within *yfcH*
R-yfcH	ATTTGCTTAGTTGTTCAGTGAGCTT	Internal region within *yfcH*
L-feoB	ACTCACGACTATTTCTGAACAGACC	Internal region within *feoB*
R-feoB	AATTGCAGCGAGAGATAAAGATTAC	Internal region within *feoB*
L-rpoSXn	CTATTTACGTACTGCAAGAGAGCTG	Internal region within *rpoS*
R-rpoSXn	GCCTTATCTGAATCACCACTAATTG	Internal region within *rpoS*
L-nilQ	GTTATCGCTGATGTAAGGAGAACAG	Internal region within *nilQ*
R-nilQ	CGGCTAGTTGCTAGTAAAATAAAGC	Internal region within *nilQ*
